# Crucial Roles of microRNA-16-5p and microRNA-27b-3p in Ameloblast Differentiation Through Regulation of Genes Associated With Amelogenesis Imperfecta

**DOI:** 10.3389/fgene.2022.788259

**Published:** 2022-03-25

**Authors:** Akiko Suzuki, Hiroki Yoshioka, Teng Liu, Aania Gull, Naina Singh, Thanh Le, Zhongming Zhao, Junichi Iwata

**Affiliations:** ^1^ Department of Diagnostic and Biomedical Sciences, School of Dentistry, The University of Texas Health Science Center at Houston, Houston, TX, United States; ^2^ Center for Craniofacial Research, The University of Texas Health Science Center at Houston, Houston, TX, United States; ^3^ Center for Precision Health, School of Biomedical Informatics, The University of Texas Health Science Center at Houston, Houston, TX, United States; ^4^ Human Genetics Center, School of Public Health, The University of Texas Health Science Center at Houston, Houston, TX, United States; ^5^ MD Anderson Cancer Center UTHealth Graduate School of Biomedical Sciences, Houston, TX, United States

**Keywords:** enamel, amelogenesis imperfecta, tooth defects, pathogenic gene, microRNA, ameloblast differentiation

## Abstract

Amelogenesis imperfecta is a congenital disorder within a heterogeneous group of conditions characterized by enamel hypoplasia. Patients suffer from early tooth loss, social embarrassment, eating difficulties, and pain due to an abnormally thin, soft, fragile, and discolored enamel with poor aesthetics and functionality. The etiology of amelogenesis imperfecta is complicated by genetic interactions. To identify mouse amelogenesis imperfecta-related genes (mAIGenes) and their respective phenotypes, we conducted a systematic literature review and database search and found and curated 70 mAIGenes across all of the databases. Our pathway enrichment analysis indicated that these genes were enriched in tooth development-associated pathways, forming four distinct groups. To explore how these genes are regulated and affect the phenotype, we predicted microRNA (miRNA)-gene interaction pairs using our bioinformatics pipeline. Our miRNA regulatory network analysis pinpointed that miR-16-5p, miR-27b-3p, and miR-23a/b-3p were hub miRNAs. The function of these hub miRNAs was evaluated through ameloblast differentiation assays with/without the candidate miRNA mimics using cultured mouse ameloblast cells. Our results revealed that overexpression of miR-16-5p and miR-27b-3p, but not miR-23a/b-3p, significantly inhibited ameloblast differentiation through regulation of mAIGenes. Thus, our study shows that miR-16-5p and miR-27b-3p are candidate pathogenic miRNAs for amelogenesis imperfecta.

## Introduction

Enamel is composed of inorganic and organic matter and water. The inorganic component, called hydroxyapatite, mainly comprises calcium, phosphate, magnesium, potassium, fluoride, and sodium, whereas the organic component includes enamel matrix proteins and enzymes. FAM20C is a Golgi-localized serine/threonine-protein kinase that is activated by FAM20A (Cui et al., 2015; Ohyama et al., 2016) and phosphorylates enamel matrix proteins, including Amelogenin (AMELX), Amelotin (AMTN), and Enamelin (ENAM), for mineralization (Ishikawa et al., 2012; Tagliabracci et al., 2012; Wang et al., 2013; Cui et al., 2015). The phosphorylated enamel matrixes provide a platform for further mineralization, during which they are cleaved and degraded by MMP20 and KLK4, and then removed from the hydroxyapatite crystals (Hu et al., 2007; Hu and Simmer, 2007). A failure in the degradation of the enamel matrixes leads to retention of enamel matrix residues between the hydroxyapatite crystals, abnormal crystal formation, and immature enamel formation (Simmer and Hu, 2002; Kwak et al., 2016; Yamazaki et al., 2019). Recent studies suggest that WDR72 may be important for the resorption of the enamel matrixes (especially for AMELX) from the extracellular matrix (ECM) through endocytosis of ameloblasts (Katsura et al., 2014; Wang et al., 2015).

Amelogenesis imperfecta (a.k.a. enamel hypoplasia) is a congenital disorder that affects the tooth surface and is characterized by abnormal enamel formation (Gadhia et al., 2012; Williams and Letra, 2018). The frequency of the condition varies among different populations worldwide, e.g., 1:700 in Sweden (Backman and Holm, 1986), 43:10,000 in Turkey (Altug-Atac and Erdem, 2007), and 1:14,000 in the United States (Crawford et al., 2007). The disorder may manifest by itself through a mutation in genes encoding enamel proteins or may accompany other morphological defects in tooth development (Aldred et al., 2003; Stephanopoulos et al., 2005; Smith et al., 2017). The affected enamel displays a wide range of severity of abnormalities, ranging from pits and grooves on the tooth’s surface to a complete loss of enamel, which results in easily brittle and worn teeth. These patients suffer from poor esthetic appearance due to tooth discoloration, abnormal tooth shape, open bite, and premature tooth loss, in addition to tooth pain, eating difficulties, and frequent and full-mouth dental maintenance and treatment (Hashem et al., 2013).

Based on the distinct phenotype and mode of inheritance, amelogenesis imperfecta can be divided into four major categories: hypoplastic enamel, hypomaturation enamel, hypocalcified enamel, and hypomature-hypoplastic enamel with taurodontism (Aldred and Crawford, 1995; Aldred et al., 2003). In hypoplastic enamel (type I), the enamel is thinner than usual but can retain its typical hardness and translucency. Due to the enamel matrix’s malfunction, the mature enamel layer often presents pits and grooves; other consequences of the thin enamel include lack of occlusion owing to small or absent cusps in the posterior molars. A distinct difference in density between dentin and the enamel layers can be seen in radiographs (Witkop, 1988; Wright, 2006). In the case of hypomaturation enamel (type II), the enamel is softer than normal due to a failure in protein removal during the maturation stage of amelogenesis. These enamel proteins that remain in the matrixes compromise the enamel matrix structure and crystal growth. While enamel thickness appears normal, its hardness is lower, resulting in pits on the surface and rapid wear. In radiographs, the enamel layer appears similar to dentin due to reduced density (Witkop, 1988; Wright, 2006). In hypocalcified enamel (type III), the enamel is softer, rougher, and more prone to rapid wear than in type II cases due to abnormal mineralization (Witkop, 1988; Urzua et al., 2011). While the enamel appears to be of normal thickness, the abnormal mineralization leads to extremely brittle teeth without a smooth and translucent appearance. The dentin in these cases is more radiopaque than the enamel (Witkop, 1988; Wright, 2006). Lastly, in the hypomature/hypoplastic enamel with taurodontism (type IV), patients have thin, pitted enamel with enlarged pulp chambers in the molars (Witkop, 1988; Wright, 2006).

Clinically, patients often present a mixed phenotype. Treatment for amelogenesis imperfecta consists in the prevention of gradual occlusal wear, in which case early detection is beneficial. Full-mouth prosthetics can preserve the remaining enamel, prevent further tooth loss, and reduce pain caused by dentin exposure (Strauch and Hahnel, 2018).

While various genetic mutations have been reported in amelogenesis imperfecta, the regulatory network remains unknown. MicroRNAs (miRNAs), typically 21–22 nucleotide long, negatively regulate gene expression at the post-transcriptional stage and usually have multiple target genes and control their expression at the regulatory network level (Guo et al., 2010; Li et al., 2020). Recent studies suggest that miRNAs play crucial roles in tooth development (Fan et al., 2015; Farmer and Mcmanus, 2017; Jin et al., 2017); therefore, this study aimed to identify the regulatory network of genes and miRNAs associated with amelogenesis imperfecta. A better understanding of the mechanism of amelogenesis imperfecta can potentially lead to the development of novel preventive and therapeutic interventions.

## Materials and Methods

### Eligibility Criteria for the Systematic Review

This systematic review followed the publishing guidelines and checklist established by PRISMA (Preferred Reporting Items for Systematic Review and Meta-Analysis). Articles were included and excluded based on the following eligibility criteria: 1) Inclusion criteria: described genes causing or potentially associated with amelogenesis imperfecta and enamel hypoplasia in species other than humans; published as original articles (not as review articles, editorials, dissertations, conference proceedings, or comments); and published in the English language; 2) Exclusion criteria: gene mutations were not described in the original articles; enamel defects resulting from exposure to environmental risk factors; cell-based experiments, molecular and biochemical analyses, structural and component analyses, and evolutional researches; and the articles failed to fit in any of the above criteria but did not include amelogenesis imperfecta candidate genes or related information.

### Information Sources and Search

The search for articles was conducted through three central literature databases: Medline (Ovid), PubMed (National Library of Medicine), and Embase (Ovid). In addition, relevant articles were searched in Scopus (Elsevier) to retrieve any studies missed in the database searches. Concepts included in the search to identify studies were *amelogenesis imperfecta* and *genetics* (gene mutation). No specific species was included in the keywords since our review included all species. A combination of Medical Subject Headings (MeSH) terms and titles, abstracts, and keywords was developed to obtain the initial Medline search string, and then adapted to the searches of the other databases. The Mouse Genome Informatics (MGI) database was searched using keywords “amelogenesis imperfecta,” “enamel hypoplasia,” “tooth enamel,” “tooth mineralization,” and “enamel mineralization” in order to provide a means of comparison and validation for the systematic review and identify genes that were potentially missed in the database searches.

### Study Selection and Data Collection

The citations searched were stored in Rayyan (https://rayyan.qcri.org/welcome), an online application for systematic reviews that stores the citations/results, automatically processes the removal of duplicates obtained through various database searches, and tracks the decisions made during the systematic review. The primary Excel workbook designed for the systematic review (http://libguides.sph.uth.tmc.edu/excel_SR_workbook) was also used for tracking search strategies and results. A Cohen’s kappa test was conducted by two screeners to check the reliability of study selection during title and abstract screening. After achieving a >90% score for the Cohen’s Kappa test, all the titles and abstracts found through the database search were full-text reviewed by the two screeners independently. All the screening results were recorded in the Primary Excel workbook, and a codebook for data collection from eligible articles was developed as previously described (Sangani et al., 2015).

### Bioinformatics Analysis

The Database for Annotation, Visualization, and Integrated Discovery (DAVID) (http://david.abcc.ncifcrf.gov/) was used for the gene set enrichment analysis. Gene Ontology (GO), including its Biological Process (BP), Molecular Function (MF), and Cellular Component (CC), and the Kyoto Encyclopedia of Genes and Genomes (KEGG) pathways were used as reference gene sets (Sun H. et al., 2019). The top five most significant pathways or GO terms were selected for further analysis. k-means was used to cluster the gene functional enrichment results and the square error to extract the closest clusters. The highly-expressed mouse tooth miRNAs were retrieved from the publications (Cao et al., 2010). The miRNA-mAIGene regulations were integrated using the data from four databases: TargetScan (version 7.1) (Agarwal et al., 2015), miRanda (August 2010 Release) (John et al., 2004), miRTarBase (Release 7.0) (Huang et al., 2020), and PITA (version 6) (Kertesz et al., 2007). Considering the possibility of false results and multiple targets for each miRNA in these databases, the intersection of miRanda and PITA was merged with the intersection of TargetScan and miRTarBase to obtain reliable miRNA-mAIGene pairs. This conservative approach was demonstrated to effectively reduce the prediction of false-positive miRNA-mAIGene pairs (Jiang et al., 2016; Bonnet et al., 2020). Each gene set (GO term or KEGG pathway) containing at least two genes was used in the core miRNA family-based regulatory network. A Fisher’s exact test was applied to assess the enrichment significance of the miRNAs. All networks were visualized using Cytoscape (Shannon et al., 2003).

### Cell Culture

The mHAT9d mouse dental epithelial cell line originated from the apical bud of the incisors was a gift from Dr. Hidemitsu Harada (Iwate Medical University, Iwate, Japan). mHAT9d cells were cultured in Dulbecco’s Modified Eagle Medium: Nutrient Mixture F-12 (DMEM/F12; Thermo Fisher Scientific) supplemented with B-27 (Thermo Fisher Scientific), 25 ng/ml basic FGF (233-FB; R&D Systems), 20 ng/ml EGF (2028-EG; R&D Systems), and penicillin/streptomycin (Otsu et al., 2016). The LS8 cell line (Chen et al., 1992) was provided by Dr. Malcolm Snead (University of Southern California). Cells were plated at a density of 60,000 cells onto a 12-well cell culture plate and maintained until 80% confluence. The cells were treated with mimic for a negative control, miR-16-5p, miR-23a-3p, miR-23b-3p, miR-27b-3p, or miR-214-3p (mirVana miRNA mimic, Thermo Fischer Scientific) using Lipofectamine RNAiMAX transfection reagent (Thermo Fisher Scientific), according to the manufacturer’s protocol (24 pmol of mimic and 3 μL of transfection reagent in 1 ml of medium per well). After 24 h of treatment, the cells at 100% confluence were cultured with differentiation medium [including 15 μg/ml retinoic acid (R2625, Sigma Aldrich) and 0.1 μM dexamethasone (D4902, Sigma Aldrich)] in order to induce ameloblast differentiation.

### Bromodeoxyuridine (BrdU) Incorporation Assay

mHAT9d cells were plated onto ibiTreat 8-well μ-slides (ibidi GmbH, Munich district, Germany) at a density of 10,000/chamber and cultured until 80% confluence. Cells were then treated with a mimic for miR-16-5p, miR-27b-3p, or control using Lipofectamine RNAiMAX transfection reagent (4.8 pmol of mimic with 0.48 µL of transfection reagent in 200 µL of proliferation medium). After 24 h of transfection, the cells were cultured under differentiation medium for 48 h. In addition, cells were treated with 100 μg/ml BrdU (Sigma Aldrich) for 1 h at day 2 of differentiation (*n* = 6 per group) and visualized with a rat monoclonal antibody against BrdU (ab6326; Abcam, 1:1,000), as previously described (Yoshioka et al., 2021a). BrdU-positive cells were quantified using images from six independent experiments.

### RNA Extraction and Quantitative Reverse Transcription-Polymerase Chain Reaction

Total RNAs were isolated from cells treated with mimics for the target miRNAs or negative control (*n* = 6 per group) using the QIAshredder and RNeasy mini extraction kit or the miRNeasy mini kit (QIAGEN), as previously described (Suzuki et al., 2019; Yan et al., 2020). In addition, total RNAs were isolated from ameloblasts at each stage of differentiation (pre-secretion, secretion, and maturation) in the lower incisors of 8-week old males C57BL/6J mice (*n* = 3). Briefly, the lower incisors were extracted, and ameloblasts were manually dissected and separated into three parts [apical 1/3 (pre-secretion), middle 1/3 (secretion), and incisal 1/3 (maturation) between the cervical loop and bony ridge of the incisor] under a dissection microscope. cDNA was reverse-transcribed with the iScript Reverse Transcription Super Mix (BioRad) and amplified with the iTaq Universal SYBER Green Super Mix (BioRad) using a CFX96 Touch Real-Time PCR Detection System (BioRad). The expression of genes was normalized with *Gapdh*. miRNA expression during ameloblast differentiation was detected with Taqman Fast Advanced Master Mix and Taqman Advanced miR cDNA Synthesis Kit (Thermo Fisher Scientific), according to the manufacturer’s instructions. The PCR primers used are listed in Supplementary Table S1.

### Immunofluorescence Analysis

The cells were plated onto ibiTreat 8-well μ-slides (ibidi GmbH, Munich district, Germany) at a density of 10,000/chamber and maintained until 80% confluency. The cells were then treated with mimics for miR-16-5p, miR-27b-3p, or a negative control, using Lipofectamine RNAiMAX transfection reagent (4.8 pmol of mimic with 0.48 µL of transfection reagent in 200 µL of differentiation medium) (*n* = 4 per group). After 24 h of treatment, the medium was replaced with differentiation medium for 2 days. AMELX expression was detected with anti-AMELX rabbit polyclonal antibody (ab153915, Abcam, 1:250), as previously described (Yoshioka et al., 2021b). Immunofluorescent images were captured with a confocal microscope (Ti-E, Nikon United States).

### Immunoblotting

The cells were plated onto 12-well plates at a density of 60,000 per well, maintained until 80% confluence, and treated with either miR-16-5p, miR-27b-3p, or a negative control mimic, for 24 h (*n* = 3 per group). The cells were then cultured in ameloblast differentiation medium for another 48 h. The treated cells were lysed with RIPA buffer (Thermo Fisher Scientific) containing a protease inhibitor cocktail (Roche) and centrifuged at 21,130 × *g* for 20 min at 4°C. The protein concentration of the supernatants was measured with the BCA protein kit (Pierce). Protein samples (30 μg) were applied to Mini-PROTEAN TGX Gels (Bio-Rad) and transferred to a polyvinylidene difluoride (PVDF) membrane. Anti-AMELX rabbit polyclonal antibody (ab153915, Abcam, 1:1,000), anti-KLK4 rabbit polyclonal antibody (PA5-109888, Thermo Fisher Scientific, 1:750), anti-MMP20 rabbit polyclonal antibody (55467-1-AP, Proteintech, 1:750), and anti-GAPDH mouse monoclonal antibody (MAB374, Millipore, 1:6,000) were used for immunoblotting. Peroxidase-conjugated anti-rabbit IgG (7074, Cell Signaling Technology, 1:100,000) and anti-mouse IgG (7076, Cell Signaling Technology, 1:100,000) were used as secondary antibodies. All immunoblotting experiments were performed three times to validate the results.

### Rescue Experiment

Cells were plated on 12-well cell culture plates at a density of 60,000 cells per well, or on ibiTreat 8-well μ-slides (ibidi GmbH, Munich district, Germany), at a density of 10,000 cells per well and maintained until 80% confluence. The cells were treated with mimics for a negative control, miR-16-5p, or miR-27b-3p (4.8 pmol for 12-well plates and 1.2 pmol for ibiTreat 8-well μ-slides) with a combination of overexpression vectors [100 ng (12-well plates) or 25 ng (ibiTreat 8-well μ-slides)] using Lipofectamine 3000 transfection reagent (Thermo Fisher Scientific), according to the manufacturer’s protocol, which was followed by treatment with *Eda* (Antibodies-online Inc., ABIN3291185), *Relt* (Antibodies-online Inc., ABIN4054001), or *Smad3* (Antibodies-online Inc., ABIN3809504) for the miR-16-5p mimic, or *Bmp2* (Antibodies-online Inc., ABIN4045152), *Pax9* (Antibodies-online Inc., ABIN4216431), or *Slc24a4* (Addgene, 75208) for the miR-27b-3p mimic (n = 6 per group). After 24 h of transfection, the medium was switched to differentiation medium for 2 days.

### Statistical Analysis

Statistical comparisons between two groups were performed with a two-tailed Student’s *t*-test. Multiple comparisons were conducted with one-way analysis of variance with the Tukey–Kramer *post hoc* test. A *p*-value of less than 0.05 was considered as statistically significant. For all groups, data were represented as mean ± SD.

## Results

### Literature and Database Search

**FIGURE 1:**
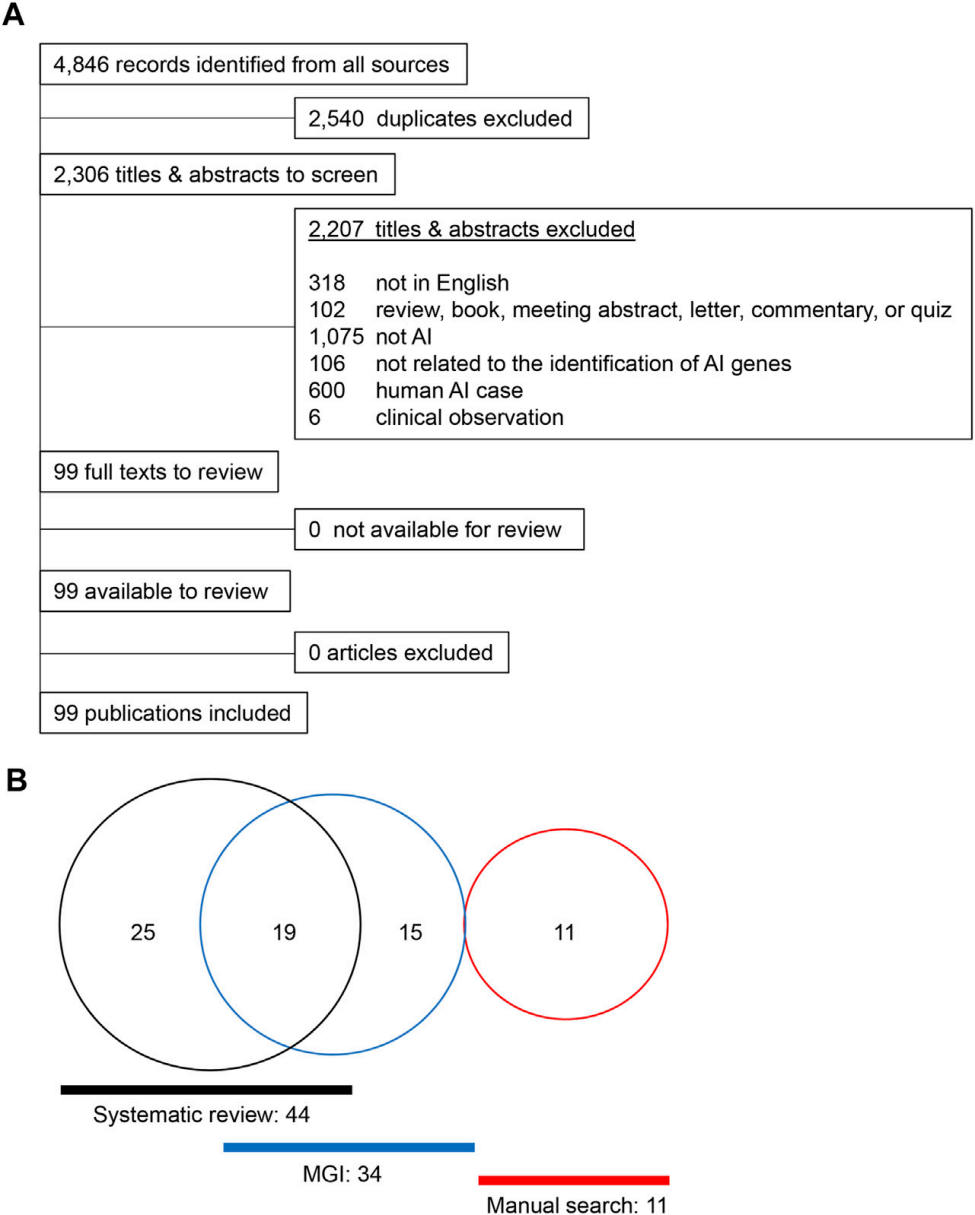
Summary of the literature search. **(A)** PRISMA flowchart for amelogenesis imperfecta articles in different species other than humans. **(B)** Venn diagram for the amelogenesis imperfecta study.

A total of 4,846 articles were extracted from a database compilation of multiple sources through a search conducted using Rayyan (Ouzzani et al., 2016). After resolving duplicates with RefWorks, 2,306 articles were selected for further screening. A total of 2,207 articles were excluded because there was no underlying genetic mechanism dictating the gene findings or the articles did not mention any relevant study or research conducted in humans. A total of 99 articles were further reviewed and qualified through a full-text review (Figure 1A), referring to 89 studies in mice, seven in rats, two in dogs, and one in cattle. A total of 44 genes [42 genes in mice with single gene mutations and two additional genes (*Bmp4* and *Stim2*) in compound mutant models] were identified in mice as genes associated with amelogenesis imperfecta through the systematic review (Supplementary Table S2). A search of the Mouse Genome Informatics (MGI) database identified a total of 59 mouse lines after the removal of duplicates. Upon validation of the enamel phenotype through review of the extracted articles, we identified 35 genes primarily associated with amelogenesis imperfecta (Supplementary Table S3). Among these 35 genes, 15 were uniquely found in the MGI search, and 19 were common in the systematic review and MGI search. Through a manual literature search, we identified additional 11 genes associated with amelogenesis imperfecta (Supplementary Table S4). As a result, a total of 70 genes were identified and curated [68 genes in single-gene mutant mice (Table 1) and two additional genes (after exclusion of overlapping genes in Table 1) in compound mutant mice (Table 2)] as genes associated with amelogenesis imperfecta (a.k.a. enamel hypoplasia) in mice (Figure 1B), hereafter referred as mouse amelogenesis imperfecta-related genes (mAIGenes). In addition, we found that three genes in rats, three genes in dogs, and one gene in cattle were reported in amelogenesis imperfecta (Supplementary Table S5). Among the 70 genes, mutations in 33 genes were reported in humans with amelogenesis imperfecta in isolated or syndromic cases.

**TABLE 1:** Single mutation mouse models with enamel defects

#	Gene Symbol	Gene Name	Location	Enamel Phenotype	Mouse Strain	PMID	Human Disease
1	*Alpl*	alkaline phosphatase, liver/bone/kidney	4 D3	hypoplastic	*Alpl* ^*−/−*^	10371245	hypophosphatasia-enamel hypoplasia
2	*Ambn*	ameloblastin	5 E1	hypoplastic	Tg (under Amelx)	12657627	isolated AI
				hypoplastic or hypocalcified	*Ambn* ^*∆5–6*^	15583034; 19375505	
				hypoplastic	*Ambn* ^*LacZ/LacZ*^	31402633	
				hypoplastic	*Ambn* ^*−/−*^	16612084	
3	*Amelx*	amelogenin, X-linked	X F5	hypoplastic	*Amelx* ^*−/−*^	11406633; 18390542; 18701811; 22243229	isolated AI
				hypomineralized	Tg (M180-∆A, M180∆A-FLAG) and Tg (M180-∆B, M180∆B-HA)	16707492; 11243888; 12619931	
				hypoplastic	Tg (M180-P70T)	17384027	
				hypoplastic	*Amelx* ^*p.Y64H/p.Y64H*^	20067920; 24363885	
				hypoplastic and hypomineralized	Tg (M194)	25117480	
				hypoplastic and hypomineralized	Tg (CTRNC)	20042744	
4	*Amtn*	amelotin	5 E1	hypomaturation and hypomineralized	*Amtn* ^*−/−*^	25715379	isolated AI
5	*Arhgap6*	Rho GTPase activating protein 6	X F5	hypoplastic	*Arhgap6* ^*−/−*^	16007484	isolated AI
6	*Ascl5* (a.k.a. *AmeloD*)	achaete-scute family bHLH transcription factor 5	1 E4	hypoplastic	*Asc5* ^*−/−*^	30504223	
7	*Bcl11b*	B cell leukemia/lymphoma 11B	12 F1	hypomineralized	*Bcl11b* ^*S826G/-*^	23727454	
8	*Bmp2*	bone morphogenetic protein 2	2 F2	hypomineralized	*Osx-Cre;Bmp2* ^*F/F*^	21597270; 25545831	
9	*Cftr*	cystic fibrosis transmembrane conductance regulator	6 A2	hypomineralized	*Cftr* ^*−/−*^	9206347; 8708137; 12161463	cystic fibrosis—AI
10	*Cldn3*	claudin 3	5 G2	hypomineralized	*Cldn3* ^*−/−*^	28596736	
11	*Cldn16*	claudin 16	16 B2	hypoplastic and hypomineralized	*Cldn16* ^*−/−*^	2642691	familial hypercalciuria and hypomagnesemia with nephrocalcinosis (FHHNC)—AI
12	*Cnnm4*	cyclin M4	1 B	hypomineralized	*Cnnm4* ^*−/−*^	24339795	Jalili syndrome—AI
13	*Col17a1*	collagen, type XVII alpha 1	19 D1	hypomaturation and hypomineralized	*Col17a1* ^*−/−*^	19036806	Junctional epidermolysis bullosa—AI
14	*Csf1* (a.k.a. *Mcsf*)	colony-stimulating factor 1 (macrophage)	3 F2	hypoplastic	OP/OP	17126805	
				hypoplastic	OP/OP; Tg (csCSF-1)	17126805	
15	*Ctnnb1*	catenin beta 1	9 F4	hypomineralized	*Amelx-Cre;Ctnnb1* ^*∆ex3F/F*^	30066216	
16	*Dlx3*	distal-less homeobox 3	11 D	hypomineralized	*K14-Cre;Dlx3* ^*F/F*^	27760456; 29745813	trichodentoosseous syndrome—AI
17	*Dmp1*	dentin matrix protein 1	5 E5	hypoplastic and hypomineralized	*Dmp1* ^*−/−*^	14966118; 14514755	hypophosphatemia—AI
18	*Dspp*	dentin sialophosphoprotein	5 E5	hypoplastic	Tg (under Amelx)	16014627	dentinogenesis imperfecta type II—AI
19	*Eda*	ectodysplasin-A	X C3	hypoplastic (no enamel)	Tg (under K14)	12812793	hypohidrotic ectodermal dysplasias not AI
20	*Enam*	enamelin	5 E1	hypomaturation	*Enam* ^*Rgsc521/Rgsc521*^	15649948; 20598351	isolated AI
				hypoplastic	*Enam* ^*Rgsc395/Rgsc395*^ *& Enam* ^*Rgsc514/Rgsc514*^	15649948	
				hypoplastic	*Enam* ^*p*^ *.* ^*Q176X/p.Q176X*^ (ATE1)	15271968; 17652207	
				hypoplastic or no enamel	*Enam* ^*LacZ/LacZ*^	18252720; 24603688	
				no enamel or hypoplastic	*Enam* ^*p.S55I/p.S55I*^ *or Enam* ^*p.S55I/+*^	28334996	
21	*Fam20a*	family with sequence similarity 20, member A	11 E1	hypoplastic and hypomineralized	*Fam20a* ^*−/−*^	22732358	enamel-renal-gingival syndrome—AI
				hypoplastic and hypomineralized	*K14-Cre;Fam20a* ^*F/F*^	27281036	
				hypoplastic (no enamel)	*Sox2-Cre;Fam20a* ^*F/F*^	31667691	
22	*Fam20c*	family with sequence similarity 20, member C	5 G2	hypoplastic (no enamel)	*Fam20c* ^*−/−*^	22732358	Raine syndrome—AI
				hypoplastic and hypomineralized	*K14-Cre;Fam20c* ^*F/F*^	24026952	
				hypoplastic and hypomineralized	*Sox2-Cre;Fam20c* ^*F/F*^	22936805	
23	*Fam83h*	family with sequence similarity 83, member H	15 D3	hypoplastic	*Fam83h* ^*−/−*^	30714208	isolated AI
				hypoplastic	Tg (truncated protein 1–296)	31060110	
24	*Fgfr1*	fibroblast growth factor receptor 1	8 A2	hypoplastic	*K14-Cre;Fgfr1* ^*F/F*^	18296607	Pfeiffer syndrome—not AI Jackson-Weiss syndrome—not AI
25	*Foxo1*	forkhead box O1	3 C	hypomaturation	*Rx-Cre;Foxo1* ^*F/F*^ & *K14-Cre;Foxo1* ^*F/F*^	22291941	
26	*Gdnf*	glial cell line derived neurotrophic factor	15 A1	hypoplastic	*Gdnf* ^*−/−*^	11878293	Hirschsprung disease type 3—not AI
27	*Gja1* (a.k.a. *Cx43*)	gap junction protein, alpha 1	10 B4	hypoplastic	*PGK-Cre;Cx43* ^*G138R/+*^	18003637	oculodentodigital dysplasia - AI
				hypoplastic	*Gja1* ^*G60S/+*^ a.k.a. *Gja1* ^*jrt/+*^	16155213; 20127707	
28	*Hmgn2*	high mobility group nucleosomal binding domain 2	4 D3	hypoplastic	Tg (under K14)	23975681	
29	*Hras*	Harvey rat sarcoma virus oncogene	7 F5	hypomineralized	*Caggs-Cre;Hras* ^*G12V/+*^	24057668; 19416908	Costello syndrome—enamel defect
30	*Irf6*	interferon regulatory factor 6	1 H6	hypoplastic	*Pitx2-Cre;Irf6* ^*F/F*^	27369589	van der Woude syndrome—not AI popliteal pterygium syndrome—not AI
31	*Itgb1*	integrin beta 1	8 E2	hypoplastic	*K14-Cre;Itgb1* ^*F/F*^	25830530	
32	*Itgb6*	integrin beta 6	2 C1.2	hypomineralized	*Itgb6* ^*−/−*^	23264742	isolated AI
33	*Klk4*	kallikrein-related peptidase 4 (prostase, enamel matrix, prostate)	7 B3	hypomineralized	*Klk4* ^*LacZ/LacZ*^	19578120	isolated AI
34	*Lama3*	laminin, alpha 3	18 A1	hypoplastic	*Lama3* ^*−/−*^	10366601	junctional epidermolysis bullosa—AI
35	*Lamb3*	laminin, beta 3	1 H6	unknown	*Lamb3* ^*Lacz/LacZ*^	27626380	junctional epidermolysis bullosa—AI
36	*Lamc2*	laminin gamma 3	1 G3	pitted enamel	Spontaneous (*Lamc2* ^*jeb*^)	20336083	cortical malformation, occipital—not AI
				hypomineralized	Tg (*TetO-Lamc2* ^*−/−*^ *;K14-rtTA;TetO-HumLAMC2*)	26956061; 23029085	
37	*Ltbp3*	latent transforming growth factor-beta binding protein 3	19 A	hypoplastic	*Ltbp3* ^*−/−*^	25669657; 28084688	dental anomalies and short stature (DASS)—AI
38	*Map3k7* (a.k.a. *Tak1*)	mitogen-activated protein kinase kinase 7	4 A5	hypomineralized	*CaMap3k7* (under Amelx)	29024853	cardiospondylocarpofacial syndrome—not AI frontometaphyseal dysplasia 2—not AI
39	*Med1*	mediator complex subunit 1	11 D	hypomineralized	*K14-Cre;Med1* ^*F/F*^	24949995; 28673966	
40	*Mmp20*	matrix metallopeptidase 20 (enamelysin)	9 A1	hypoplastic	*Mmp20* ^*−/−*^	12393861; 15557396; 24466234	isolated AI
				hypomineralized	Tg (under Amelx)	24466234; 29481294	
41	*Msx2*	msh homeobox 2	13 B1	hypoplastic	*Msx2* ^*LacZ/LacZ*^	20934968; 17878071	isolated AI enlarged parietal foramina 1—not AI craniosynostosis type 2 - not AI
42	*Nectin1*	nectin cell adhesion molecule 1	9 A5	hypomineralized	*Nectin1* ^*−/−*^	18703497; 21038445	cleft lip and palate/ectodermal dysplasia 1—not AI
43	*Nectin3*	nectin cell adhesion molecule 3	16 B5	unknown	*Nectin3* ^*−/−*^	21038445	
44	*Pax9*	paired box 9	12 C1	hypoplastic	*Pax9* ^*neo/neo*^	16236760	tooth agenesis, selective, 3—not AI
45	*Plau* (a.k.a. *uPA*)	plasminogen activator, urokinase	14 A3	unknown-chalky white	Tg (under K5)	9927592; 15161662	
46	*Pitx2*	paired-like homeodomain transcription factor 2	3 G3	unknown	*Pitx2* ^*−/−*^	27626380	Axenfeld-Rieger syndrome—not AI iridogoniodysgenesis syndrome - not AI Peters anomaly—not AI
47	*Postn*	periostin, osteoblast-specific factor	3 C	unknown-chalky white	*Postn* ^*LacZ/LacZ*^	16314533	
				unknown-chalky white but thick enamel	*Postn* ^*−/−*^	16497272	
48	*Rac1*	Rac family small GTPase 1	5 G2	hypoplastic and hypomineralized	*K14-Cre;Rac1* ^*F/F*^	22243243	mental retardation, autosomal dominant, 48—not AI
49	*Relt*	RELT tumor necrosis factor receptor	7 E2	hypomineralized	*Relt* ^p.P390*/p.P390*^	30506946	isolated AI
50	*Rhoa*	ras homolog family member A	9 F1-F2	hypoplastic	Tg (dominant-negative, under Amelx)	21576911; 23841780	
51	*Runx1*	runt-related transcription factor 1	16 C4	hypoplastic	*K14-Cre;Runx1* ^*F/F*^	30026553	Braddock-Carey syndrome (BCS)—AI
52	*Runx2*	runt-related transcription factor 2	17 B3	hypomineralized	*K14-Cre;Runx2* ^*F/F*^	29941908	metaphyseal dysplasia with maxillary hypoplasia and brachydactyly—AI cleidocranial dysplasia—not AI
53	*Slc4a4*	solute carrier family 4 (anion exchanger), member 4	5 E1	hypoplastic and hypomineralized	*Slc4a4* ^*−/−*^	20529845; 25012520	proximal renal tubular acidosis—AI
54	*Slc10a7*	solute carrier family 10 (sodium/bile acid cotransporter family), member 7	8 C1	hypoplastic	*Slc10a7* ^*−/−*^	30082715	skeletal dysplasia—AI
				hypomaturation and hypomineralized	*Slc10a7* ^*−/−*^	30082715	
55	*Slc12a2*	solute carrier family 12, member 2	18 D3	hypomineralized	*Slc12a2* ^*−/−*^	29209227	
56	*Slc13a5*	solute carrier family 13 (sodium-dependent citrate transporter), member 5	11 B4	hypoplastic	*Slc13a5* ^*−/−*^	28406943	Kohlschütter-Tönz syndrome (KTS)—AI early infantile epileptic encephalopathy 25 (EIEE25)-tooth hypoplasia and hypodontia—not AI
57	*Slc24a4*	solute carrier family 24 (sodium/potassium/calcium exchanger), member 4	12 E	hypomineralized	*Slc24a4* ^*−/−*^	23375655	isolated AI
58	*Smad3*	SMAD family member 3	9 C	hypomineralized	*Smad3* ^*−/−*^	12763048	Loeys-Dietz syndrome—not AI
59	*Sp3*	trans-acting transcription factor 3	2 C3	hypoplastic (no enamel)	*Sp3* ^*−/−*^	10675334	
60	*Sp6*	trans-acting transcription factor 6	11 D	hypoplastic	*Sp6* ^*−/−*^	30504223; 18156176; 18297738	
61	*Sp7* (a.k.a. *Osx*)	trans-acting transcription factor 7 (osterix)	15 F3	unknown (die at birth)	*Sp7* ^*−/−*^	29405385	osteogenesis imperfecta type XII - not AI
62	*Stim1*	stromal interaction molecule 1	7 E2-E3	hypomineralized	*K14-Cre;Stim1* ^*F/F*^	28732182	AI tubular aggregate myopathy—not AI Stormorken syndrome—not AI
				hypoplastic and hypomineralized	*Amelx-Cre;Stim1* ^*F/F*^	31329049	
63	*Tbx1*	T-box 1	16 A3	hypoplastic (no enamel)	*Tbx1* ^*−/−*^	19233155	22q-11.2 deletion syndrome (DiGeorge syndrome)—AI
64	*Tcirg1* (a.k.a. *ATP6a3*)	T cell, immune regulator 1, ATPase, H+ transporting, lysosomal V0 protein A3	19 A	hypomineralized	spontaneous	23174213	autosomal recessive osteopetrosis—not AI
65	*Tgfb1*	transforming growth factor, beta 1	7 A3	hypoplastic	Tg (under Dspp)	16674659; 11116156	Camurati-Engelmann disease—not AI
				hypomineralized	*Tgfb1* ^*Tgfb3/Tgfb3*^	24056369	
				hypomineralized	*K14-Cre;Tgb1* ^*F/F*^	30243146	
66	*Tgfbr2*	transforming growth factor, beta receptor II	9 F3	hypoplastic and hypomineralized	*Amelx-Cre;Tgfbr2* ^*F/F*^	24278477	Loeys-Dietz syndrome—not AI familial thoracic aortic aneurysm and dissection - not AI
67	*Tmbim6*	transmembrane BAX inhibitor motif containing 6	15 F1	hypomineralized	*Tmbim6* ^*−/−*^	30963569	
68	*Wdr72*	WD repeat domain 72	9 D	hypomaturation and hypomineralized	*Wdr72* ^*LacZ/LacZ*^	25008349; 26247047	isolated AI

AI: amelogenesis imperfecta; OP: osteopetrotic; Tg: transgenic.

**TABLE 2:** Compound mutant mouse models with enamel defects.

#	Gene Symbol	Gene Name	Location	Enamel Phenotype	Mouse Strain	PMID
1	*Ambn* and *Enam*	ameloblastin and enamelin	5 E1 and 5 E1	hypoplastic	*Ambn* ^*+/-*^ *;Enam* ^*+/-*^	31478359
2	*Bmp2* and *Bmp4*	bone morphogenetic protein 2 & bone morphogenetic protein 4	2 F2 and 14 C4	hypomineralized	*K14-Cre;Bmp2* ^*F/F*^ *;Bmp4* ^*F*^ */* ^*F*^	27146352
3	*Klk4* and *Mmp20*	kallikrein related-peptidase 4 and matrix metallopeptidase 20	7 B3 and 9 A1	hypoplastic and hypomineralized	*Klk4* ^*−/−*^ *;Mmp20* ^*−/−*^	27066511
4	*Stim1* and *Stim2*	stromal interaction molecule 1 and stromal interaction molecule 2	7 E2-E3 and 5 C1	hypomineralized	*K14-Cre;Stim1* ^*F/F*^ *;Stim2* ^*F/F*^	28732182

These mAIGenes were further categorized into three classes of amelogenesis imperfecta based on gross anatomical observation, histological analysis, microCT, and component analyses, which all are established in human cases: hypoplastic/enamel hypoplasia/no enamel (40 genes), hypomaturation (6 genes), hypomineralized/hypocalcified (39 genes), and unknown detailed classification (4 genes) (Table 3). Some genes exhibited a combined phenotype, as seen in humans. It should be noted that different mutational strategies for deletion, overexpression, or knock-in of the same gene sometimes resulted in different tooth phenotypes. This suggests that subtle changes in the expression or deletion of non-coding genomic sequences may affect the expression and function of genes that are crucial for enamel formation.

**TABLE 3:** Classification of enamel defects.

Phenotype	Gene Symbols
hypoplastic/no enamel/chalky-white	*Alpl*, *Ambn*, *Amelx*, *Arhgap6*, *Ascl5*, *Cldn16*, *Csf1*, *Dmp1*, *Dspp*, *Eda*, *Enam*, *Fam20a*, *Fam20c*, *Fam83h*, *Fgfr1*, *Gdnf*, *Gja1*, *Hmgn2*, *Itgb1*, *Irf6*, *Lama3*, *Ltbp3*, *Mmp20*, *Msx2*, *Pax9*, *Plau*, *Postn*, *Rac1*, *Rhoa*, *Runx1*, *Slc4a4*, *Slc13a5*, *Sp3*, *Sp6*, *Stim1*, *Tbx1*, *Tgfb1*, *Tgfbr2*, *Ambn* and *Enam*, *Klk4* & *Mmp20*
hypomaturation	*Amtn*, *Col17a1*, *Enam*, *Foxo1*, *Slc10a7*, *Wdr72*
hypomineralized/hypocalcified	*Amelx*, *Amtn*, *Bcl11b*, *Bmp2*, *Cftr*, *Cldn3*, *Cldn16*, *Cnnm4*, *Col17a1*, *Ctnnb1*, *Dlx3*, *Dmp1*, *Fam20a*, *Fam20c*, *Hras*, *Itgb6*, *Klk4*, *Lamc2*, *Map3k7*, *Med1*, *Mmp20*, *Nectin1*, *Rac1*, *Relt*, *Runx2*, *Smad3*, *Slc4a4*, *Slc10a7*, *Slc12a2*, *Slc24a4*, *Stim1*, *Tcirg1*, *Tgfb1*, *Tgfbr2*, *Tmbim6*, *Wdr72*, *Bmp2* & *Bmp4*, *Klk4* & *Mmp20*, *Stim1* & *Stim2*
unknown	*Lamb3*, *Nectin3*, *Pitx2*, *Sp7*

Among the mAIGenes, 12 genes (*Ambn*, *Amelx*, *Amtn*, *Col17a1*, *Csf1*, *Dmp1*, *Dspp*, *Enam*, *Lama3*, *Lamb3*, *Lamc2*, and *Postn*) were grouped in the extracellular matrix (ECM) pathway, 11 genes (*Alpl*, *Fam20a*, *Fam20c*, *Hras*, *Klk4*, *Map3k7*, *Mmp20*, *Plau*, *Rac1*, *Rhoa*, and *Tcirg1*) in the enzyme pathway, and seven genes (*Cftr*, *Cnnm4*, *Slc4a4*, *Slc10a7*, *Slc12a2*, *Slc13a5*, and *Slc24a4*) in the ion exchanger/transporter pathway. Moreover, three genes (*Stim1*, *Stim2*, and *Tmbim6*) were related to a calcium ion sensor or regulator, and six genes (*Cldn3*, *Cldn16*, *Ctnnb1*, *Gja1*, *Nectin1*, and *Nectin3*) were involved in cell-cell or cell-ECM adhesions. Since ameloblasts secrete enamel proteins, mutations in genes related to ECM and enamel proteins support their causal roles in amelogenesis imperfecta. In addition, a substantial number of genes were involved in growth factor signaling cascades: four were growth factors (*Bmp2*, *Bmp4*, *Gdnf*, and *Tgfb1*), five receptors (*Fgfr1*, *Itgb1*, *Itgb6*, *Relt*, and *Tgfbr2*), 15 transcription factors (*Ascl5*, *Bcl11b*, *Ctnnb1*, *Dlx3*, *Foxo1*, *Irf6*, *Msx2*, *Pax9*, *Pitx2*, *Runx1*, *Runx2*, *Sp3*, *Sp6*, *Sp7*, and *Tbx1*), two transcriptional regulators (*Hmgn2* and *Med1*), and one a signal mediator (*Smad3*). Since these factors are involved in various developmental processes, the mutations would be related to syndromic cases with various developmental defects beyond amelogenesis imperfecta (Table 4).

**TABLE 4:** Functional category of amelogenesis imperfecta-related genes.

Category Name	Gene Symbols
Extracellular matrix	*Ambn*, *Amelx*, *Amtn*, *Col17a1*, *Csf1*, *Dmp1*, *Dspp*, *Enam*, *Lama3, Lamb3*, *Lamc2*, *Postn*
Enzyme	*Alpl*, *Fam20a*, *Fam20c*, *Hras*, *Klk4*, *Map3k7*, *Mmp20*, *Plau*, *Rac1*, *Rhoa*, *Tcirg1*
Receptor	*Fgfr1*, *Itgb1*, *Itgb6*, *Relt*, *Tgfbr2*
Receptor binding molecule	*Ltbp3*
Ion exchanger or transporter	*Cftr*, *Cnnm4*, *Slc4a4*, *Slc10a7*, *Slc12a2*, *Slc13a5*, *Slc24a4*
Calcium sensor or regulator	*Stim1*, *Stim2*, *Tmbim6*
Cell-cell or cell-ECM adhesion molecule	*Cldn3*, *Cldn16*, *Ctnnb1*, *Gja1*, *Nectin1*, *Nectin3*
Growth factor	*Bmp2*, *Bmp4*, *Gdnf*, *Tgfb1*
Transcriptional factor	*Ascl5*, *Bcl11b*, *Ctnnb1*, *Dlx3*, *Foxo1*, *Irf6*, *Msx2*, *Pax9*, *Pitx2*, *Runx1*, *Runx2*, *Sp3*, *Sp6*, *Sp7*, *Tbx1*
Transcriptional regulator	*Hmgn2*, *Med1*
Signal mediator	*Smad3*
Unknown	*Fam83h*, *Wrd72*

### Functional Enrichment Analysis of mAIGenes

**FIGURE 2:**
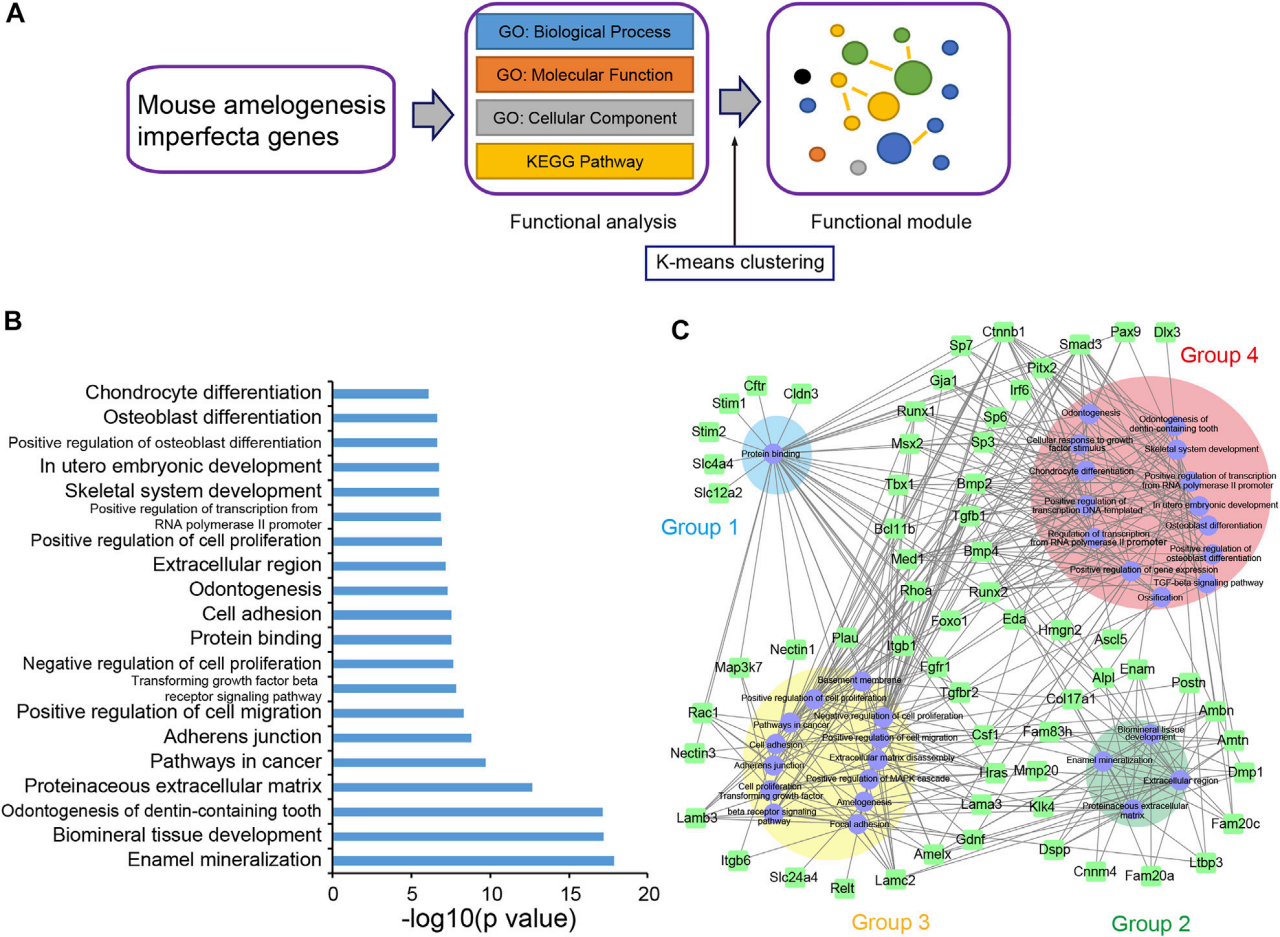
Functional enrichment analysis of mouse amelogenesis imperfecta-related genes (mAIGenes). **(A)** Flowchart of the functional enrichment analysis. Significant Gene Ontology (GO) terms and KEGG pathways were determined by a false discovery rate (FDR) < 0.01. The significant gene sets were then clustered into functional modules using a k-means clustering algorithm. **(B)** Top 20 GO terms or KEGG pathways. Gene sets related to tooth development and cell proliferation were among the top enriched sets. **(C)** The gene set network showed four groups, which were ordered by the number of gene sets (the smallest number of gene sets was in Group 1). These gene sets were related to protein banding, enamel mineralization, cancer, and bone development, respectively.

To further explore the functional features of mAIGenes, we performed a functional enrichment analysis and functional module cluster analysis (Figure 2A). Using a false discovery rate (FDR) < 0.01, we obtained 32 gene sets that were significantly enriched in mAIGenes, including four pathways from Kyoto Encyclopedia of Genes and Genomes (KEGG) annotations, 24 Gene Ontology (GO) Biological Process (BP) terms, three GO Cellular Component (CC) terms, and one GO Molecular Function (MF) term (Table 5). Among the top 20 most significant gene sets, genes associated with tooth development (e.g., enamel mineralization, biomineral tissue development, and odontogenesis of dentin-containing tooth) were among the most significantly enriched (Figure 2B). To investigate how these functional terms and pathways are interrelated, we used the k-means algorithm to cluster them (Supplementary Figure S1A). This analysis revealed four groups (Table 5), including the 32 gene sets mentioned above and 63 mAIGenes in the module network (Figure 2C). The groups were ordered by number of gene set, with the smaller number being named first. Group 1 had one gene set— “Protein binding” —, which included *Stim1*, *Stim2*, *Slc4a4*, *Slc12a2*, etc. (Figure 2C), whereas Group 2 was related to biomineral development and ECM. These two pathways are closely related, since most of the biomineral development process occurs in extracellular fluids (Figure 2C). *Enam*, *Ambn*, *Amtn*, and *Amelx* were commonly involved in biomineralization during tooth enamel development and located at the ECM (Figure 2C). Cell proliferation and cancer-related gene sets were clustered in Group 3, including “Cell proliferation”, “Pathways in cancer”, “Cell adhesion”, and “Positive regulation of cell migration” (Figure 2C). Group 4 highly reflected the tooth and bone development, as it contained “Odontogenesis of biomineral tissue development”, “Odontogenesis of dentin-containing tooth”, “Ossification”, “Osteoblast differentiation”, and “Skeletal system development” (Figure 2C). *Bmp2*, *Bmp4*, and *Runx2* connected most of the gene sets in Group 4 (Supplementary Figure S1B), and these genes have been reported to play critical roles in bone development.

**TABLE 5:** Top functional enrichment clusters.

Pathway	Cluster #
positive regulation of cell migration	1
transforming growth factor-beta receptor signaling pathway	1
growth factor activity	1
transforming growth factor-beta receptor binding	1
TGF-beta signaling pathway	1
apical junction complex	2
cell adhesion molecule binding	2
adherens junction	2
colorectal cancer	2
basement membrane	3
laminin-5 complex	3
pathways in cancer	3
focal adhesion	3
enamel mineralization	4
biomineral tissue development	4
odontogenesis of dentin-containing tooth	4
structural constituent of tooth enamel	4
proteinaceous extracellular matrix	5
extracellular region	5
protein binding	6

### miRNA-mAIGene Regulatory Network and Identification of Critical miRNAs

**FIGURE 3:**
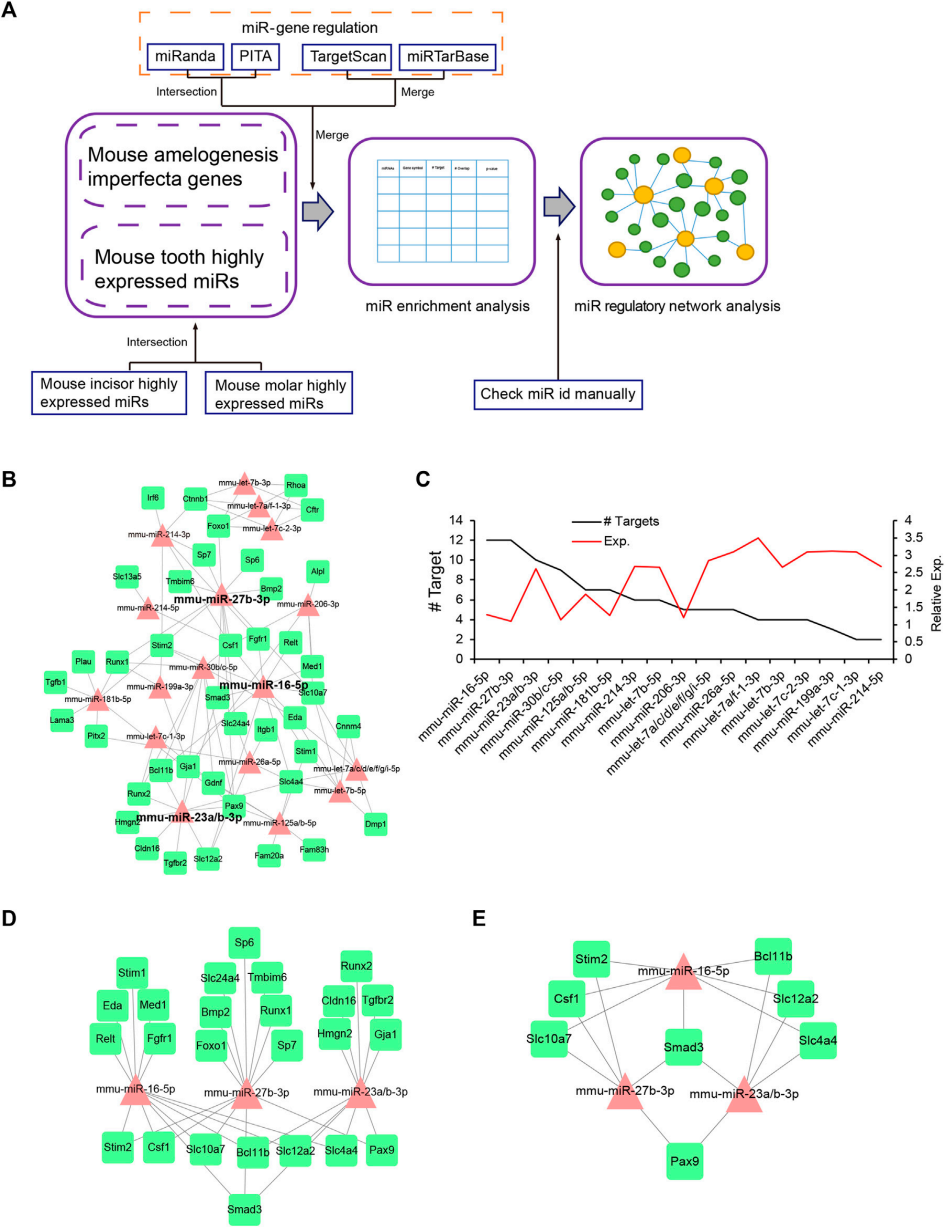
miRNA-mAIGene regulatory network and features. **(A)** Flowchart of the miRNA regulatory network analysis. The miRNA-mAIGene pairs were first identified using four miR-target databases with adjusted *p*-value < 0.05. Next, the miRNA regulatory network analysis was performed. **(B)** The miRNA regulatory network, which included 17 miRNAs, 41 mAIGenes, and 103 miRNA-mAIGene pairs. Three miRNAs (i.e., miR-16-5p, miR-27b-3p, and miR-23a/b-3p) were the hub miRNAs in the network. **(C)** Degree distribution of the miRNAs in the miRNA-mAIGene regulatory network in B, with miR-16-5p, miR-27b-3p, and miR-23a/b-3p having the highest degrees. **(D)** The sub-network of miR-16-5p, miR-27b-3p, and miR-23a/b-3p. **(E)** The sub-network of genes regulating more than two miRNAs in Figure 3D *.*

For the miRNA-mAIGene regulatory network analysis, we performed miRNA-mAIGene enrichment analysis and miRNA regulatory network analysis (Figure 3A). We identified 35 Highly Expressed MiRNAs (HEMs) in mouse incisors and 32 HEMs in molars with a frequency >1%; 26 mouse tooth HEMs were then curated by taking the intersection of the incisor and molar HEMs (Supplementary Table S6) [26]. A total of 21 of these HEMs did not have a confident -3p or -5p; therefore, we considered that these had both -3p and -5p and identified 47 HEMs, all with a certain -3p or -5p. Based on these 47 HEMs, we predicted that 32 HEMs might target the 42 mAIGenes by using our pipeline and the four miRNA-target gene databases: TargetScan, miRanda, miRTarBase, and PITA. By performing the miRNA-mAIGene regulatory relationship enrichment analysis with a cutoff adjusted *p*-value < 0.05, we identified 27 notable miRNAs, 41 genes, and 161 miRNA-mAIGene pairs. A total of 17 miRNAs or miRNA groups, 41 genes, and 103 miRNA-mAIGene pairs were extracted after merging the miRNAs or miRNA groups that shared the same targets (such as miR-23a/b-3p and miR-125a/b-5p) (Table 6). Three miRNAs (miR-16-5p, miR-27b-3p, and miR-23a/b-3p) were considered to be hubs in the miRNA regulatory network (Figure 3B) because they had the highest degrees (Figure 3C, Supplementary Table S7), which are defined as the number of partners that immediately interact with a node of interest in the network (Sun et al., 2012), and the lowest adjusted *p*-values (Table 6). The sub-network of miR-16-5p, miR-27b-3p, and miR-23a/b-3p showed that Smad3 was regulated by all the three hub miRNAs. *Stim2*, *Csf1*, *Slc10a7*, *Bcl11b*, *Slc12a2*, *Slc12a2*, *Slc4a4*, and *Pax9* were regulated by two of these three hub miRNAs or miRNA group, whereas the other genes were regulated by one miRNA or miRNA group (Figures 3D,E). As above, miR-16-5p, miR-27b-5p, and miR-23a/b-3p were considered to be promising miRNA candidates for amelogenesis imperfecta in mice.

**TABLE 6:** MicroRNA (miRNA) enrichment analysis of mouse genes related to amelogenesis imperfecta.

miR ID	Target Genes	# Targets	Adjusted *p*-value	FDR
miR-16-5p	*Bcl11b*, *Csf1*, *Eda*, *Fgfr1*, *Med1*, *Relt*, *Slc4a4*, *Slc10a7*, *Slc12a2*, *Smad3*, *Stim1*, *Stim2*	12	3.94 × 10^–7^	1.63 × 10^–3^
miR-27b-3p	*Bmp2*, *Csf1*, *Foxo1*, *Pax9*, *Runx1*, *Slc10a7*, *Slc24a4*, *Smad3*, *Sp6*, *Sp7*, *Stim2*, *Tmbim6*	12	4.12 × 10^–7^	1.69 × 10^–3^
miR-23a/b-3p	*Bcl11b*, *Cldn16*, *Gja1*, *Hmgn2*, *Pax9*, *Runx2*, *Slc4a4*, *Slc12a2*, *Smad3*, *Tgfbr2*	10	8.51 × 10^–6^	2.01 × 10^–3^
miR-214-3p	*Csf1*, *Ctnnb1*, *Fgfr1*, *Irf6*, *Sp7*, *Stim2*	6	9.38 × 10^–5^	1.43 × 10^–3^
miR-30b/c-5p	*Bcl11b*, *Csf1*, *Eda*, *Gdnf*, *Gja1*, *Pax9*, *Runx1*, *Runx2*, *Stim2*	9	3.82 × 10^–4^	4.49 × 10^–3^
miR-125a/b-5p	*Fam20a*, *Fam83h*, *Gdnf*, *Gja1*, *Pax9*, *Slc4a4*, *Stim1*	7	7.57 × 10^–4^	7.86 × 10^–3^
let-7a/f-1-3p	*Cftr*, *Ctnnb1*, *Foxo1*, *Rhoa*	4	9.98 × 10^–4^	9.85 × 10^–3^
let-7b-3p	*Cftr*, *Ctnnb1*, *Foxo1*, *Rhoa*	4	9.98 × 10^–4^	9.85 × 10^–3^
let-7c-2-3p	*Cftr*, *Ctnnb1*, *Foxo1*, *Rhoa*	4	9.98 × 10^–4^	9.85 × 10^–3^
miR-181b-5p	*Lama3*, *Pax9*, *Plau*, *Pitx2*, *Runx1*, *Stim2*, *Tgfb1*	7	3.03 × 10^–3^	2.45 × 10^–2^
miR-206-3p	*Alpl*, *Csf1*, *Gja1*, *Med1*, *Slc10a7*	5	8.07 × 10^–3^	5.53 × 10^–2^
let-7c-1-3p	*Gdnf*, *Runx2*	2	1.29 × 10^–2^	8.20 × 10^–2^
let-7b-5p	*Cnnm4*, *Dmp1*, *Eda*, *Slc4a4*, *Slc10a7*, *Stim1*	6	1.77 × 10^–2^	1.07 × 10^–1^
miR-199a-3p	*Gja1*, *Runx1*, *Stim2*	3	3.40 × 10^–2^	1.89 × 10^–1^
let-7a/c/d/e/f/g/i-5p	*Cnnm4*, *Dmp1*, *Eda*, *Slc4a4*, *Slc10a7*	5	3.43 × 10^–2^	1.90 × 10^–1^
miR-214-5p	*Csf1*, *Slc13a5*	2	3.92 × 10^–2^	2.14 × 10^–1^
miR-26a-5p	*Itgb1*, *Pitx2*, *Slc4a4*, *Slc12a2*, *Slc24a4*	5	4.91 × 10^–2^	2.62 × 10^–1^

Adjusted *p*-value < 0.05 was used as the cutoff threshold. FDR: false discovery rate. miRNAs sharing the same target genes and with the same adjusted *p*-value were merged (e.g., miR-23a/b-3p).

### Experimental Validation

**FIGURE 4:**
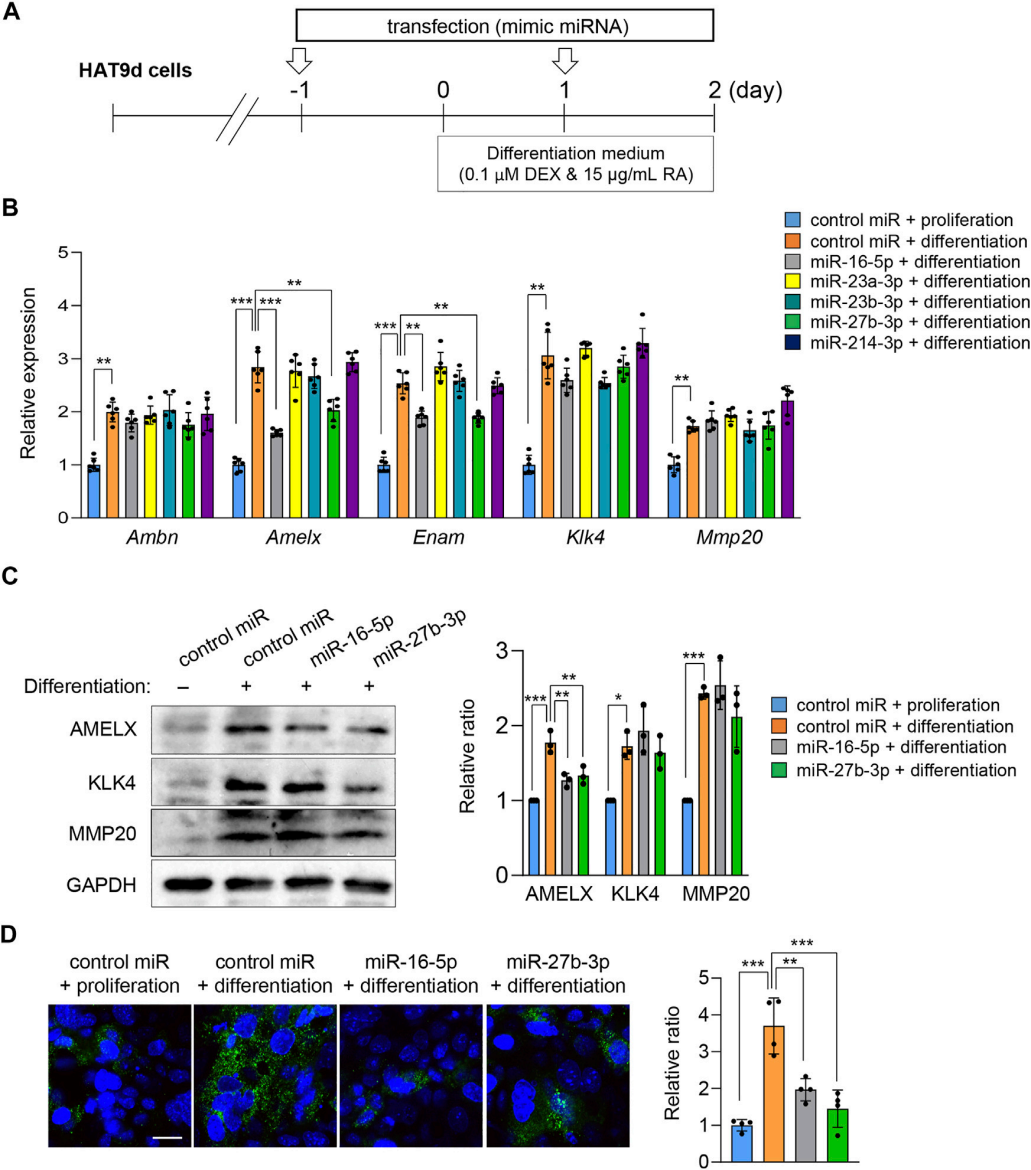
Effects of overexpression of candidate miRNAs asssociated with amelogenesis imperfecta on ameloblast differentiation. **(A)** Schematic of the experiment. **(B)** Gene expression of the indicated genes after treatment with the mimic for the indicated miRNA in mHAT9d cells (*n* = 6). **p < 0.01; ***p < 0.001. **(C)** Immunoblotting for AMELX, KLK4, MMP20, and GAPDH (internal control) in mHAT9d cells under the indicated conditions. Graph shows the quantification of the immunoblotting. *n* = 3 per group. *p < 0.05; **p < 0.01; ***p < 0.001. **(D)** ICC for AMELX in mHAT9d cells under the indicated conditions. Scale bar, 50 μm. Graph shows the quantification of images from four independent experiments. **p < 0.01; ***p < 0.001.

To evaluate the function of the miRNAs predicted by the bioinformatic analyses, we conducted ameloblast differentiation assays using mHAT9d cells, a mouse dental epithelial cell line. Although the mouse ameloblast-like cells LS8 (Chen et al., 1992) have been widely used for ameloblast studies, they are limited in their ability to differentiate. We analyzed both LS8 and mHAT9d cells under differentiation conditions and found that mHAT9d cells reacted better to the induction of differentiation (Figure 4A). For instance, the expression of the ameloblast differentiation maker genes was induced more strongly in mHAT9d cells compared to LS8 cells (Supplementary Figures S2, S3). Therefore, mHAT9d cells were used in this study. We found that expression of ameloblast differentiation marker genes (i.e., *Ambn*, *Amelx*, *Enam*, *Klk4,* and *Mmp20*) was induced with ameloblast differentiation medium (Figure 4B, Supplementary Figure S3). In addition, we tested whether other genes associated with amelogenesis imperfecta were induced. Among the 27 genes regulated by miR-16-5p, miR-23a-3p, miR-23b-3p, miR-27b-3p, and miR-214-3p, we found 14 genes that were upregulated under differentiation conditions (Supplementary Figure S4). miR-16-5p and miR-27b-3p were induced at relatively high expression levels in mHAT9d cells, and their expression did not change under differentiation conditions (Supplementary Figure S5A). In addition, we found that miR-16-5p and miR-27b-3p were expressed at the pre-secretion, secretion, and maturation stages of ameloblast differentiation in mouse lower incisors (Supplementary Figure S5B). Overexpression of either miR-16-5p or miR-27b-3p significantly anti-correlated with downregulation of expression of *Amelx* and *Enam,* but not *Ambn*, *Klk4*, and *Mmp20*, in mHAT9d cells (Figure 4B). We confirmed that the expression levels of AMELX, but not KLK4 and MMP20, were decreased by overexpression of miR-16-5p and miR-27b-3p with immunoblotting (Figure 4C). The expression of AMELX was further confirmed by immunocytochemical analysis (Figure 4D). By contrast, mimics for miR-23a-3p, miR-23b-3p, and miR-214-3p did not affect the gene expression of the ameloblast differentiation makers (Figure 4B). These results indicate that miR-16-5p and miR-27b-3p may play a critical role in ameloblast differentiation through the regulation of genes that are crucial for ameloblast differentiation.

**FIGURE 5:**
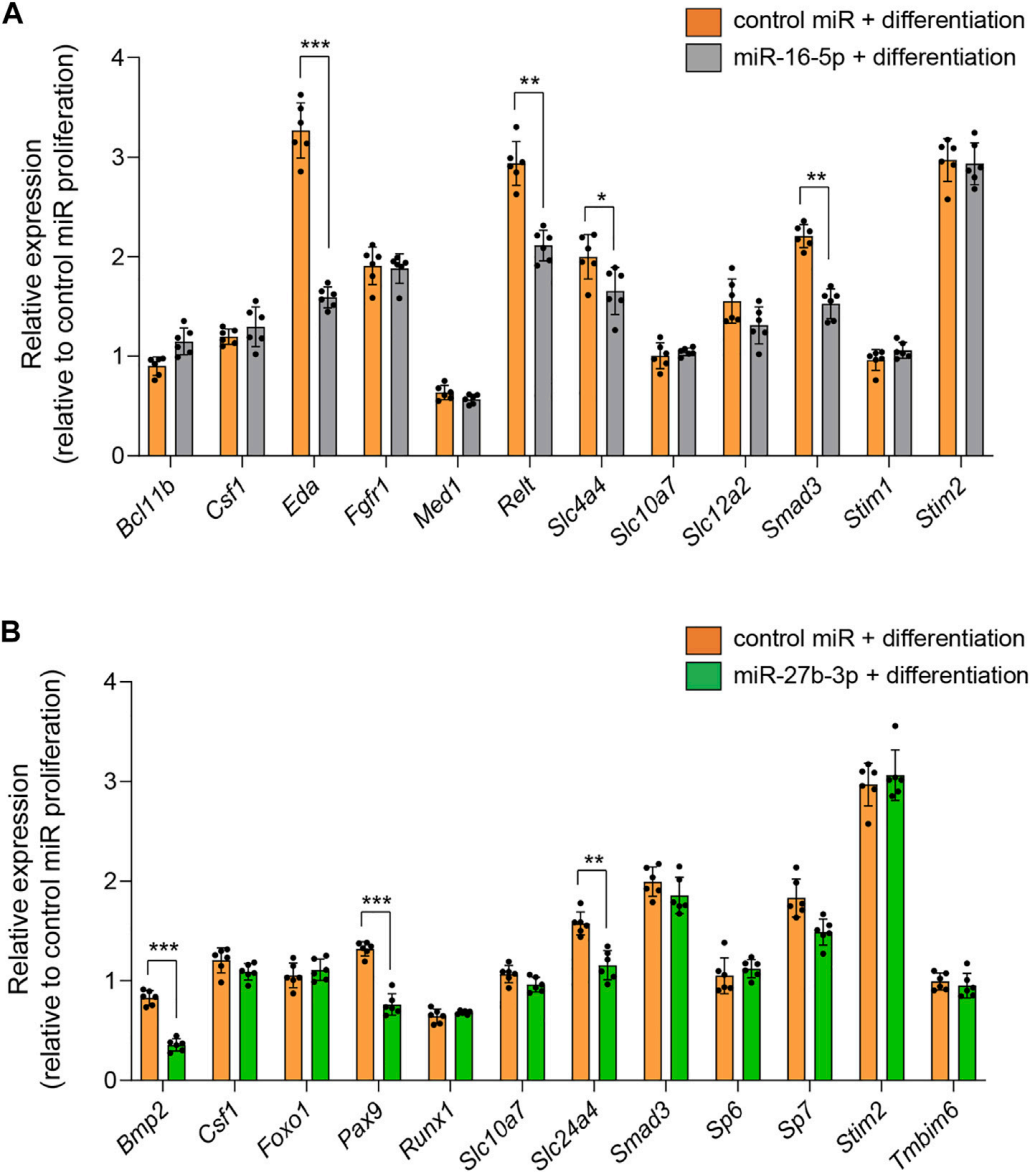
Effects of overexpression of miR-16-5p and miR-27b-3p on expression of target genes. **(A)** Quantitative RT-PCR analyses for target genes after treatment with control and miR-16-5p mimic under differentiation conditions (*n* = 6). *p < 0.05; **p < 0.01; ***p < 0.001. **(B)** Quantitative RT-PCR analyses for target genes after treatment with control and miR-27b-3p mimic under differentiation conditions (*n* = 6). **p < 0.01; ***p < 0.001.

Next, to identify the miRNA-mAIGene regulatory mechanism(s), we conducted quantitative RT-PCR (qRT-PCR) analyses for the predicted target genes for each miRNA (*Bcl11b*, *Csf1*, *Eda*, *Fgfr1*, *Med1*, *Relt*, *Slc4a4*, *Slc10a7*, *Slc12a2*, *Smad3*, *Stim1*, and *Stim2* for miR-16-5p; *Bmp2*, *Csf1*, *Foxo1*, *Pax9*, *Runx1*, *Slc10a7*, *Slc24a4*, *Smad3*, *Sp6, Sp7*, *Stim2*, and *Tmbim6* for miR-27b-3p) in mHAT9d cells. The expression of *Eda*, *Relt*, *Slc4a4*, and *Smad3* was significantly downregulated in mHAT9d cells treated with miR-16-5p mimic (Figure 5A, Supplementary Figure S6). Similarly, the expression of *Bmp2*, *Pax9*, and *Slc24a4* was significantly downregulated in mHAT9d cells treated with miR-27b-3p mimic (Figure 5B, Supplementary Figure S6). Furthermore, we confirmed that treatment of inhibitor for either miR-16-5p or miR-27b-3p had no effect on expression of *Amelx* and *Enam*, while the expression of the target genes of each miRNA was upregulated (Supplementary Figure S7). Indeed, the predicted target genes contained miRNA recognition sites for their correlated miRNAs on the 3′-UTR (Supplementary Figure S8). By contrast, there was no potential recognition site for miR-16-5p on *Amelx* and *Enam* and for miR-27b-3p on *Amelx*, while there was a potential recognition site for miR-27b-3p on *Enam*, and treatment with either mimic or inhibitor for miR-16-5p and miR-27b-3p failed to alter the expression of *Amelx* and *Enam*, suggesting that these genes are indirectly regulated by miR-16-5p and miR-27b-3p in mHAT9d cells.

**FIGURE 6:**
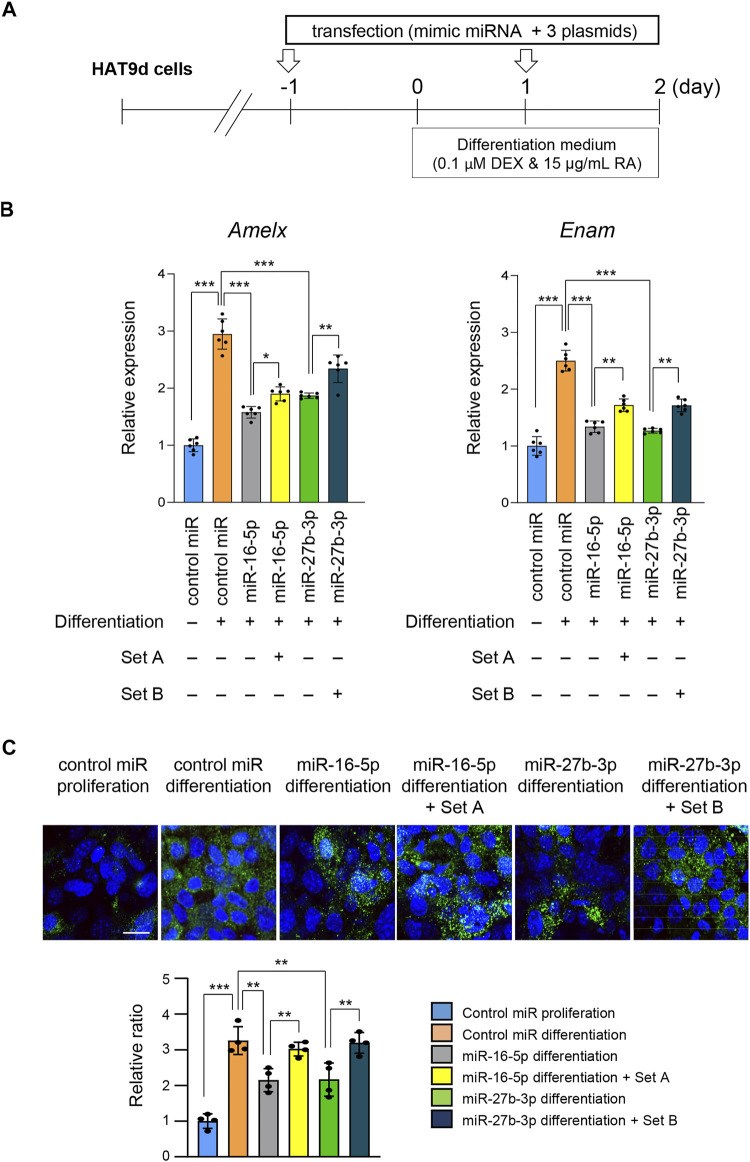
Overexpression of target genes following overexpression of miR-16-5p and miR-27b-3p. **(A)** Schematic of the experiment. **(B)** Gene expression of Amelx and Enam following overexpression of Eda, Relt, and Smad3 under overexpression of control and miR-16-5p mimic, or of Bmp2, Pax9, and Slc24a4 under overexpression of control and miR-27b-3p mimic, in mHAT9d cells (*n* = 6). *p < 0.05; **p < 0.01; ***p < 0.001. **(C)** ICC for AMELX in mHAT9d cells under the indicated conditions. Scale bar, 50 μm. Graph shows the quantification of images from four independent experiments. **p < 0.01; ***p < 0.001.

Finally, to examine the functional relevance of genes that were significantly downregulated under treatment with either miR-16-5p or miR-27b-3p mimic, we conducted rescue experiments by overexpressing the target genes (Figure 6A). We found that overexpression of *Eda, Relt,* and *Smad3* under conditions of overexpression of miR-16-5p partially restored mRNA and protein expression of *Amelx* and *Enam* (Figures 6B,C). Similarly, overexpression of *Bmp2*, *Pax9*, and *Slc24a4* partially restored mRNA and protein expression of *Amelx* and *Enam* when miR-27b-3p was overexpressed (Figures 6B,C). Taken together, our results show that overexpression of miR-16-5p and miR-27b-3p inhibits ameloblast differentiation through the regulation of *mAIGenes*.

## Discussion

This study aimed to identify regulatory networks for the genes and miRNAs involved in amelogenesis imperfecta in mouse models. Through a literature and MGI searches, we identified 70 genes associated with ameloblast imperfecta and predicted 27 miRNAs to be involved in the development of amelogenesis imperfecta in mice. We found that overexpression of miR-16-5p and miR-27b-3p in mHAT9d cells suppresses *Amelx* and *Enam* under ameloblast differentiation conditions, respectively.

In this study, we found that overexpression of miR-16-5p inhibited expression of *Eda*, *Relt*, *Slc4a4*, and *Smad3*. miR-16-5p has been detected in osteosarcoma, osteoarthritis, and bone fracture healing. Its overexpression induces suppression of *SMAD3*, resulting in inhibition of cell proliferation, migration, and invasion in osteosarcoma cells (Gu et al., 2020), and in downregulation of *COL2A1* and *Aggrecan* and upregulation of *ADAMTS* in chondrocytes, which may be involved in the development of osteoarthritis (Li et al., 2015). In addition, overexpression of miR-16-5p suppresses *BACH2* in gingival epithelial cells and *Bcl2* and *Ccnd1* in MC3T3-E1 cells, resulting in apoptosis and G1/S cell cycle arrest (Sun Y. et al., 2019; Liu et al., 2020).

RELT, a TNF receptor superfamily, is cleaved at the extracellular domain by ADAM10, a metalloprotease that is expressed at the apical loop during the transition stage of ameloblasts (Ikeda et al., 2019). ADAM10 also cleaves type XVII collagen, a component of the basement membrane (Franzke et al., 2009). Mice deficient for either *Relt* or *Col17a1* display a hypomineralized enamel defect (Asaka et al., 2009; Kim et al., 2019). Currently, no mutations in *ADAM10* have been reported in amelogenesis imperfecta in humans and mice; therefore, the role of ADAM10 in amelogenesis imperfecta is unclear.

EDA is a TNF family transmembrane protein that binds to its receptor EDAR and initiates NF-κB signaling. Overexpression of *Eda* in mice results in hypoplastic amelogenesis imperfecta (Mustonen et al., 2003); in humans, mutations in either *EDA* or *EDAR* have been found in hypohidrotic ectodermal dysplasia and isolated tooth agenesis, but not in amelogenesis imperfecta (Shen et al., 2019; Wright et al., 2019; Yu et al., 2019; Andreoni et al., 2021).

SLC4A4, a sodium bicarbonate co-transporter (NBCe1), is involved in the regulation of bicarbonate transportation and intracellular pH homeostasis (Bernardo et al., 2006; Urzua et al., 2011). Mice deficient for *Slc4a4* exhibit hypomineralized amelogenesis imperfecta; therefore, NBCe1 is responsible for a change in extracellular pH during enamel maturation (Lacruz et al., 2010; Jalali et al., 2014).

SMAD3 transduces canonical TGF-β signals together with SMAD2 and SMAD4 in the regulation of downstream genes under developmental and pathological conditions. *Smad3* knockout mice exhibit hypomineralized amelogenesis imperfecta through downregulation of genes involved in biomineralization (e.g., *Ambn*, *Amel*, *Enam*, *Mmp20*, *Klk4*, and *Gja1*) (Yokozeki et al., 2003; Poche et al., 2012).

In addition, we found that overexpression of miR-27b-3p inhibits expression of *Bmp2*, *Pax9*, and *Slc24a4*. Previous studies suggest that overexpression of miR-27b-3p in stem cells in the bone marrow or the maxillary sinus membrane suppresses osteogenic differentiation via suppression of *KDM4B* or *Sp7*, respectively (Peng et al., 2017; Zhang et al., 2020). Moreover, miR-27b-3p is downregulated in cartilage in patients with rheumatoid arthritis compared to healthy individuals. In chondrocytes, overexpression of miR-27b-3p suppresses Caspase-3 and upregulates BCL-2, resulting in apoptosis inhibition (Zhou et al., 2019).

BMP2 is a TGF-β superfamily growth factor involved in the development and homeostasis of mineral tissues (Chen et al., 2004; Halloran et al., 2020). Mice with a deletion of *Bmp2* in osteogenic and odontogenic cells (*Osx-Cre;Bmp2* ^F/F^ cKO) exhibit hypomineralized amelogenesis imperfecta and incisal malocclusion through downregulation of *Enam*, *Amelx*, *Mmp20*, and *Klk4* (Feng et al., 2011; Guo et al., 2015). Moreover, mice with an odontoblast-specific deletion of *Bmp2* (*Dmp1-Cre;Bmp2* and *Wnt1-Cre;Bmp2* cKO) show dentinogenesis imperfecta without enamel formation defects (Jani et al., 2018; Malik et al., 2018).

PAX9, a transcription factor, plays a role in craniofacial and skeletal development, including the development of tooth, bone, cartilage, and muscle (Monsoro-Burq, 2015; Farley-Barnes et al., 2020). Several single nucleotide polymorphisms (SNPs) in *PAX9* are reported to be associated with tooth size and shape as well as tooth agenesis (Lee et al., 2012; Wong et al., 2018; Safari et al., 2020; Alkhatib et al., 2021). While *Pax9* null mice exhibit cleft palate and tooth developmental arrest at the bud stage (Zhou et al., 2011), hypomorphic *Pax9* mutant mice exhibit hypoplastic amelogenesis imperfecta in the lower incisors and tooth agenesis of the third molars (Kist et al., 2005).

SLC24A4, a potassium-dependent sodium/calcium exchanger (NCKX4), is expressed in ameloblasts at the maturation stage and plays an important role in calcium ion transport by exchanging intracellular Ca^2+^ and K^+^ with extracellular Na^2+^ for Ca^2+^ supply into the developing enamel crystals (Hu et al., 2012; Bronckers et al., 2015). A deficiency of *Slc24a4* causes hypomineralized amelogenesis imperfecta in mice (Parry et al., 2013), and mutations in *SLC24A4* are associated with isolated amelogenesis imperfecta (either hypomineralized or hypomaturation types) in humans (Parry et al., 2013; Seymen et al., 2014; Herzog et al., 2015; Khan et al., 2020).

Our results from the rescue experiments suggest that miR-16-5p and miR-27b-3p are involved in amelogenesis imperfecta through dysregulation of mAIGenes. In summary, our systematic search for mAIGenes provides an overview of the genes involved in this condition. Our bioinformatics pipeline identified three potential miRNAs that may actively interact with mAIGenes, and two of these miRNAs were experimentally validated in mouse cell lines. These results will expand our knowledge of the genetics of amelogenesis imperfecta in animal models, which can be translated into human studies and help develop clinical approaches for diagnosis and treatment. We will need to further evaluate the functional significance of these miRNA-gene regulatory networks *in vivo*. Both the negative and positive feedback loops between the miRNAs and target genes should also be further evaluated in various cell lines and *in vivo* since miRNAs may regulate the expression of multiple genes and multiple miRNAs may regulate the expression of a single gene. In addition, transcription factors may be involved in these miRNA-gene regulatory networks; for example, a direct regulation between miRNA and mAIGenes may be bypassed through other transcription factors.

## Data Availability

The original contributions presented in the study are included in the article/Supplementary Material, further inquiries can be directed to the corresponding authors.
