# Supplementary material for: Crucial Roles of microRNA-16-5p and microRNA-27b-3p in Ameloblast Differentiation Through Regulation of Genes Associated With Amelogenesis Imperfecta
Source: Front Genet. 2022 Mar 25;13:788259. doi: 10.3389/fgene.2022.788259 (PMC8990915; doi:10.3389/fgene.2022.788259)

Supplementary Material

Supplementary Data

Supplementary Figure 1. mAIGene functional enrichment square error analysis and sub-network visualization. (**A**) Plot for square error vs k value. The square error (SE) difference between two neighboring k values was used to estimate the best k value. $[SE(k) – SE(k-1)] \geq 20$ was applied as the cutoff for our dataset, and 4 was the best k value. (**B**) Sub-network of *Bmp2*, *Bmp4,* and *Runx2* from the functional module network.

Supplementary Figure 2. Ameloblast differentiation in cultured LS8 cells. (**A**) Schematic of the experiment. (**B**) Quantitative RT-PCR analyses for the indicated genes under treatment with 5, 10, or 20 μg/mL retinoic acid and 0.1 μM dexamethasone at day 2 (n = 3). $*p < 0.05$; $**p < 0.01$; $***p < 0.001$ vs control.

Supplementary Figure 3. Ameloblast differentiation in cultured mHAT9d cells. (**A**) Quantitative RT-PCR analyses for the indicated genes under treatment with 0.5, 1, 5, 10, or 15 μg/mL retinoic acid and 0.1 μM dexamethasone at day 2 (n = 3). $*p < 0.05$; $**p < 0.01$; $***p < 0.001$ vs control. (**B**) Quantitative RT-PCR analyses for the indicated genes at day 0, 2, and 4 under ameloblast differentiation conditions (15 μg/mL retinoic acid and 0.1 μM dexamethasone) (n = 3). $**p < 0.01$; $***p < 0.001$ vs control. (**C**) BrdU staining at day 2 under ameloblast differentiation conditions. Scale bar, 50 μm. (**D**) Quantification of BrdU-positive cells in C.

Supplementary Figure 4. Ameloblast differentiation in cultured mHAT9d cells. Quantitative RT-PCR analyses of the indicated genes at day 2 under ameloblast differentiation conditions (15 μg/mL retinoic acid and 0.1 μM dexamethasone) (n = 3). $*p < 0.05$; $**p < 0.01$; $***p < 0.001$ vs control.

Supplementary Figure 5. Expression of miR-16-5p, miR-27b-3p, and their target genes in mHAT9d cells and mouse lower incisors. (**A**) Expression of miR-16-5p and miR-27b-3p under proliferation and ameloblast differentiation (Day 2) conditions (15 μg/mL retinoic acid and 0.1 μM dexamethasone) compared to miR-26a-5p (a housekeeping control miRNA) (n = 3). (**B**) Expression of miR-16-5p and miR-27b-3p in ameloblasts at pre-secretion, secretion, and maturation stages in mouse lower incisors compared to miR-26a-5p. $*p < 0.05$ (n = 3). (**C**) Expression of *Bmp2*, *Pax9*, *Relt*, and *Smad3* in ameloblasts at pre-secretion, secretion, and maturation stages in mouse lower incisors. $*p < 0.05$; $**p < 0.01$ (n = 3).

Supplementary Figure 6. Transfection efficiency of miRNA mimics for miR-16-5p and miR-27b-3p. (**A**) Schematic of the experiment. (**B**) miR-16-5p expression after treatment with the mimic in mHAT9d cells (n = 3). $**p < 0.01$. (**C**) miR-27b-3p expression after treatment with the mimic in mHAT9d cells (n = 3). $***p < 0.001$.

Supplementary Figure 7. Effects of inhibitor of miR-16-5p and miR-27b-3p on gene expression. (**A**) Schematic of the experiment. (**B**) miR-16-5p expression after treatment with the inhibitor in mHAT9d cells (n = 3). ***$p < 0.001$. (**C**) miR-27b-3p expression after treatment with the inhibitor in mHAT9d cells (n = 3). ***$p < 0.001$. (**D**) Quantitative RT-PCR for *Amelx* and *Enam* after treatment with an inhbiitor of either control, miR-16-5p, or miR-27b-3p under proliferation and differentiation conditions (n = 3). **$p < 0.01$; ***$p < 0.001$. (**E**) Quantitative RT-PCR for the indicated genes after treatment with a control or miR-16-5p inhibitor under differentiation conditions (n = 3). **$p < 0.01$; ***$p < 0.001$. (**F**) Quantitative RT-PCR for the indicated genes after treatment with a control or miR-27b-3p inhibitor under differentiation conditions (n = 3). *$p < 0.05$; **$p < 0.01$.

Supplementary Figure 8. Putative target sites on target genes for miR-16-5p and miR-27b-3p. (**A**) Bioinformatic analysis of the complementarity of the miR-16-5p seed-sequence to the 3' UTR of the indicated genes. (**B**) Bioinformatic analysis of the complementarity of the miR-27b-3p seed-sequence to the 3' UTR of the indicated genes. (**C**) Quantitatve RT-PCR for *Amelx* and *Enam* after treatment with either mimic (left) or inhibitor (right) for control, miR-16-5p, and miR-27b-3p (n = 3).

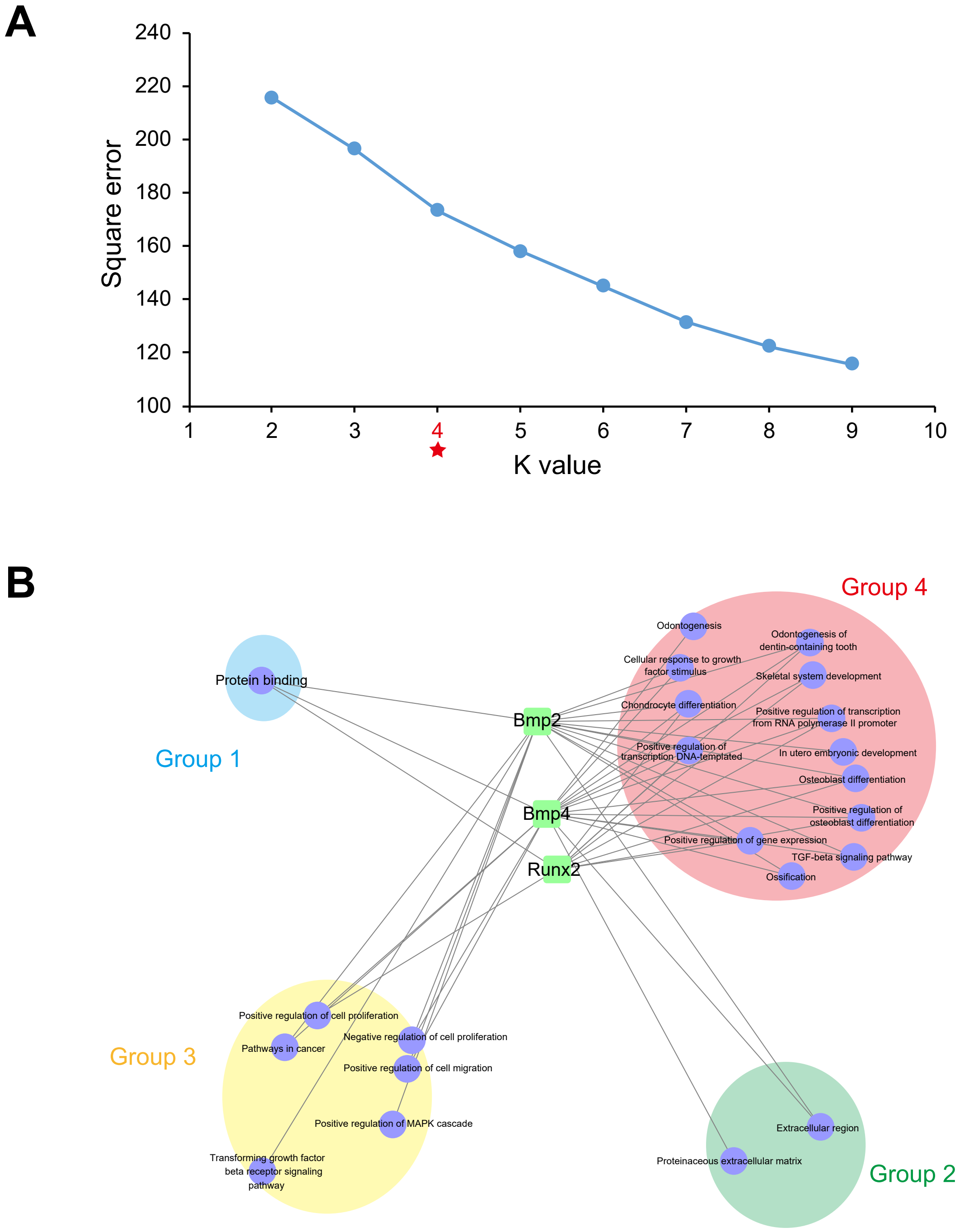
A
Square error
240
220
200
180
160
140
120
100
1
2
3
4
5
6
7
8
9
10
K value
B
Group 1
Group 2
Group 3
Group 4
Protein binding
Bmp2
Bmp4
Runx2
Odontogenesis
Odontogenesis of dentin-containing tooth
Cellular response to growth factor stimulus
Skeletal system development
Chondrocyte differentiation
Positive regulation of transcription from RNA polymerase II promoter
Positive regulation of transcription DNA-templated
In utero embryonic development
Osteoblast differentiation
Positive regulation of osteoblast differentiation
Positive regulation of gene expression
TGF-beta signaling pathway
Ossification
Positive regulation of cell proliferation
Pathways in cancer
Negative regulation of cell proliferation
Positive regulation of cell migration
Positive regulation of MAPK cascade
Transforming growth factor beta receptor signaling pathway
Extracellular region
Proteinaceous extracellular matrix

Supplementary Figure 2

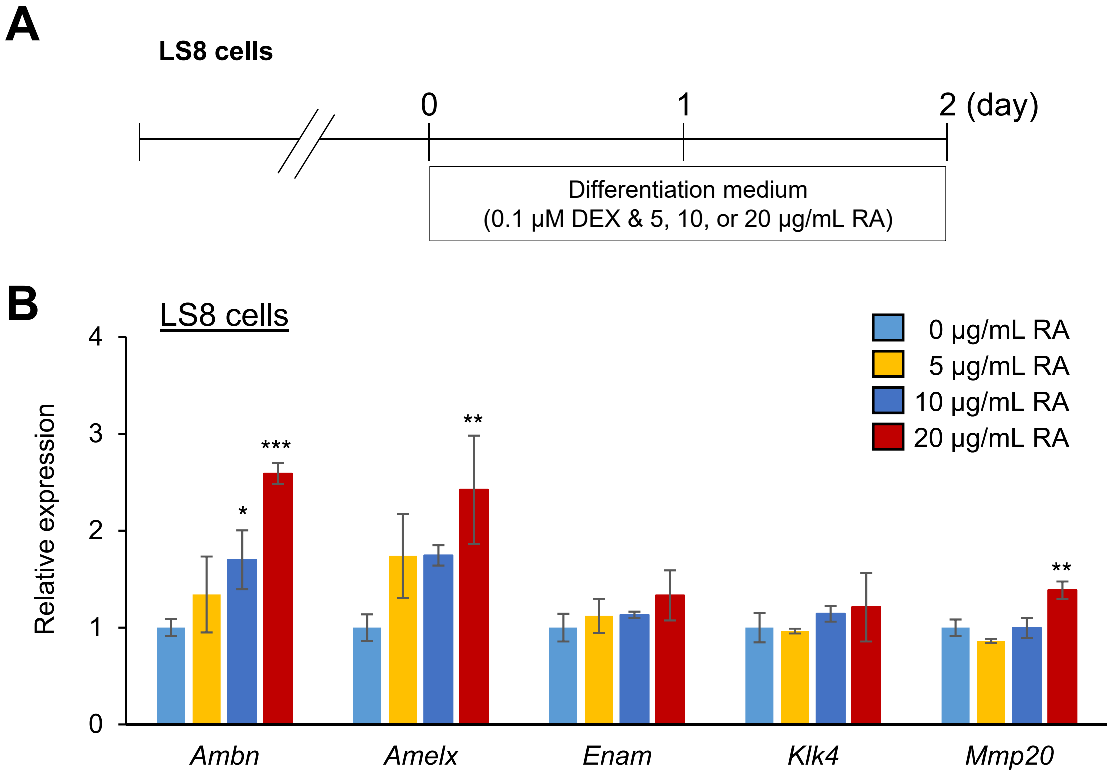

Supplementary Figure 3

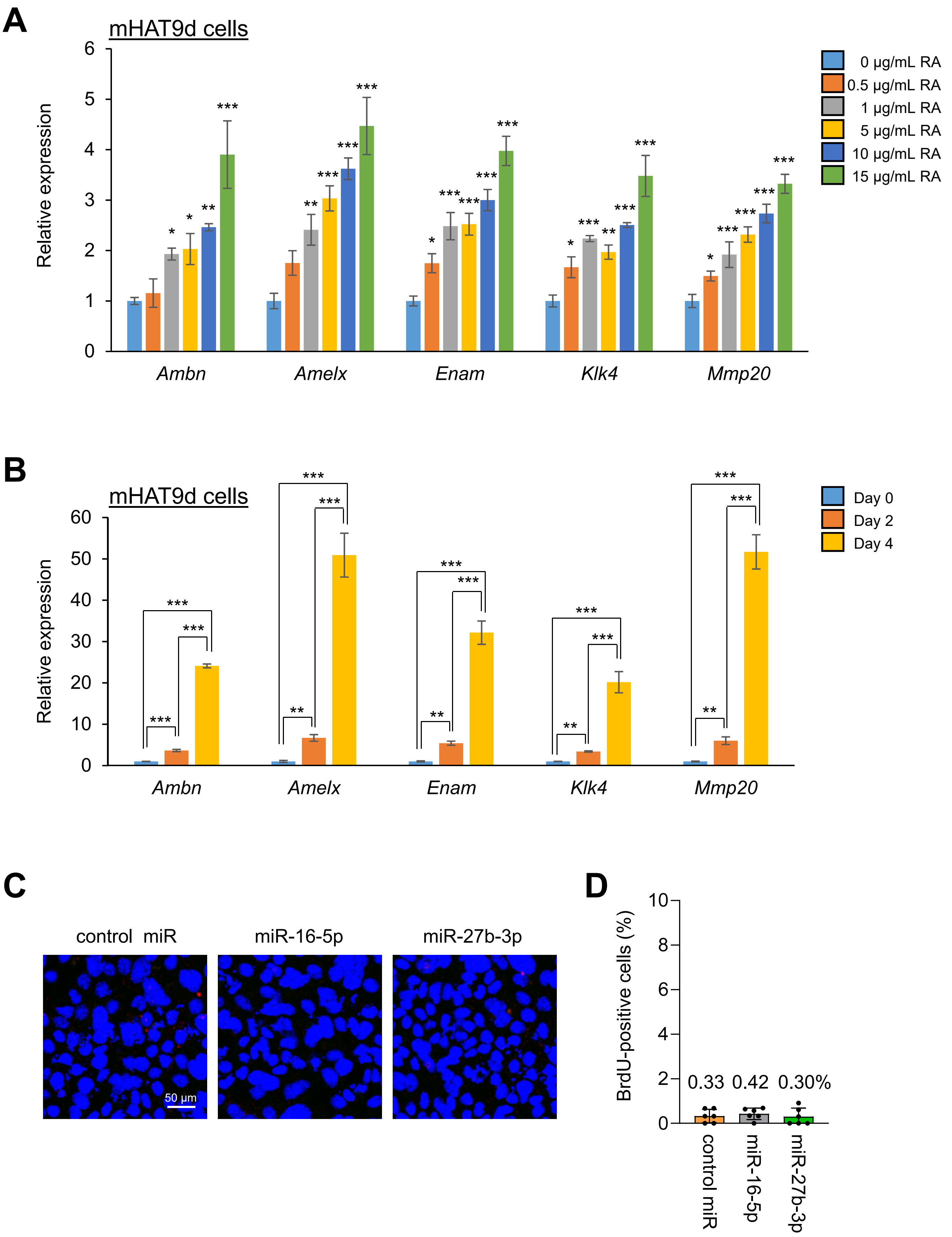

Supplementary Figure 4

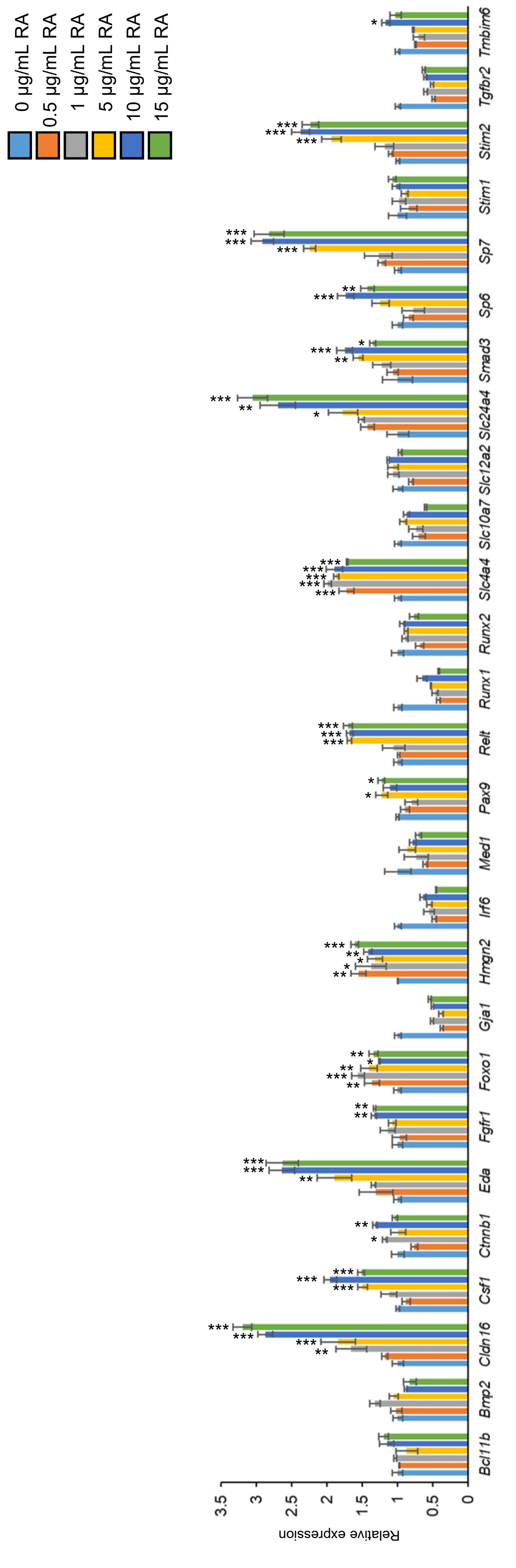

Supplementary Figure 5

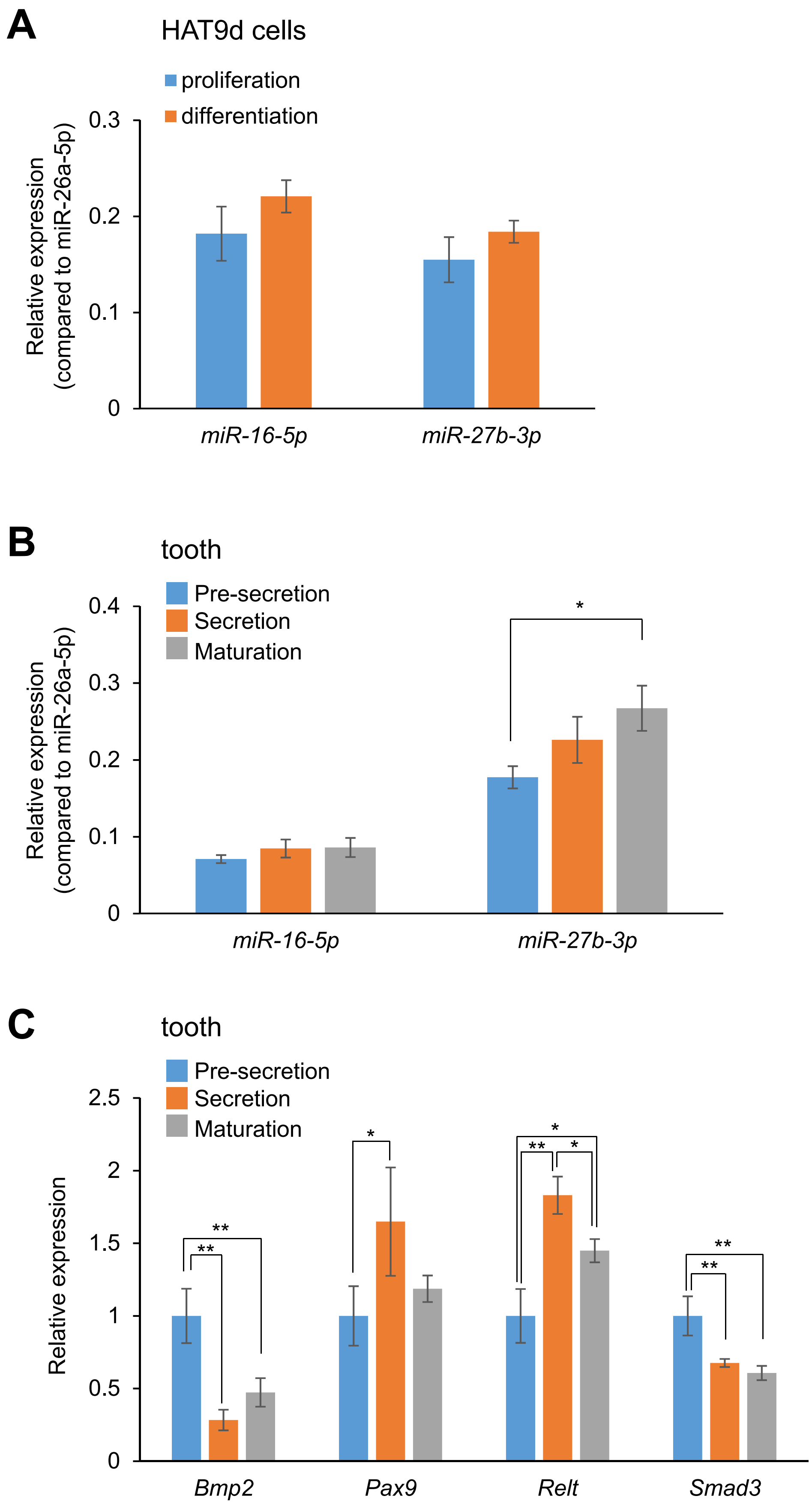

Supplementary Figure 6

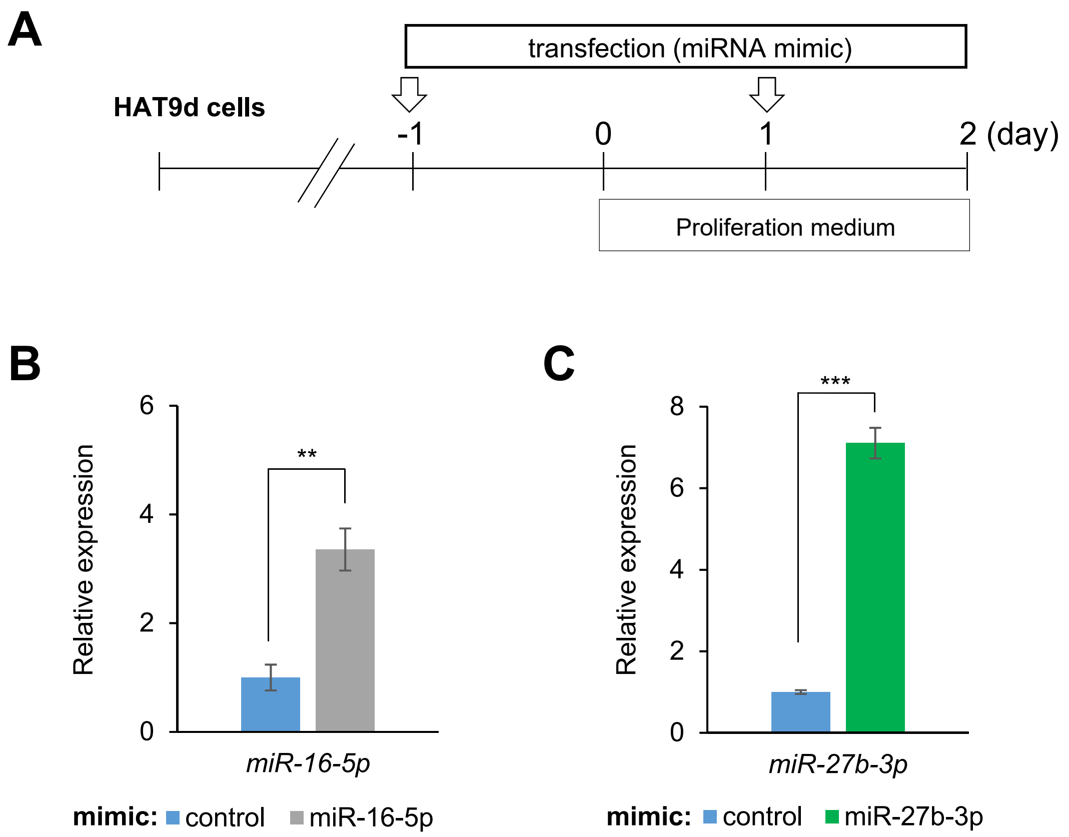

Supplementary Figure 7

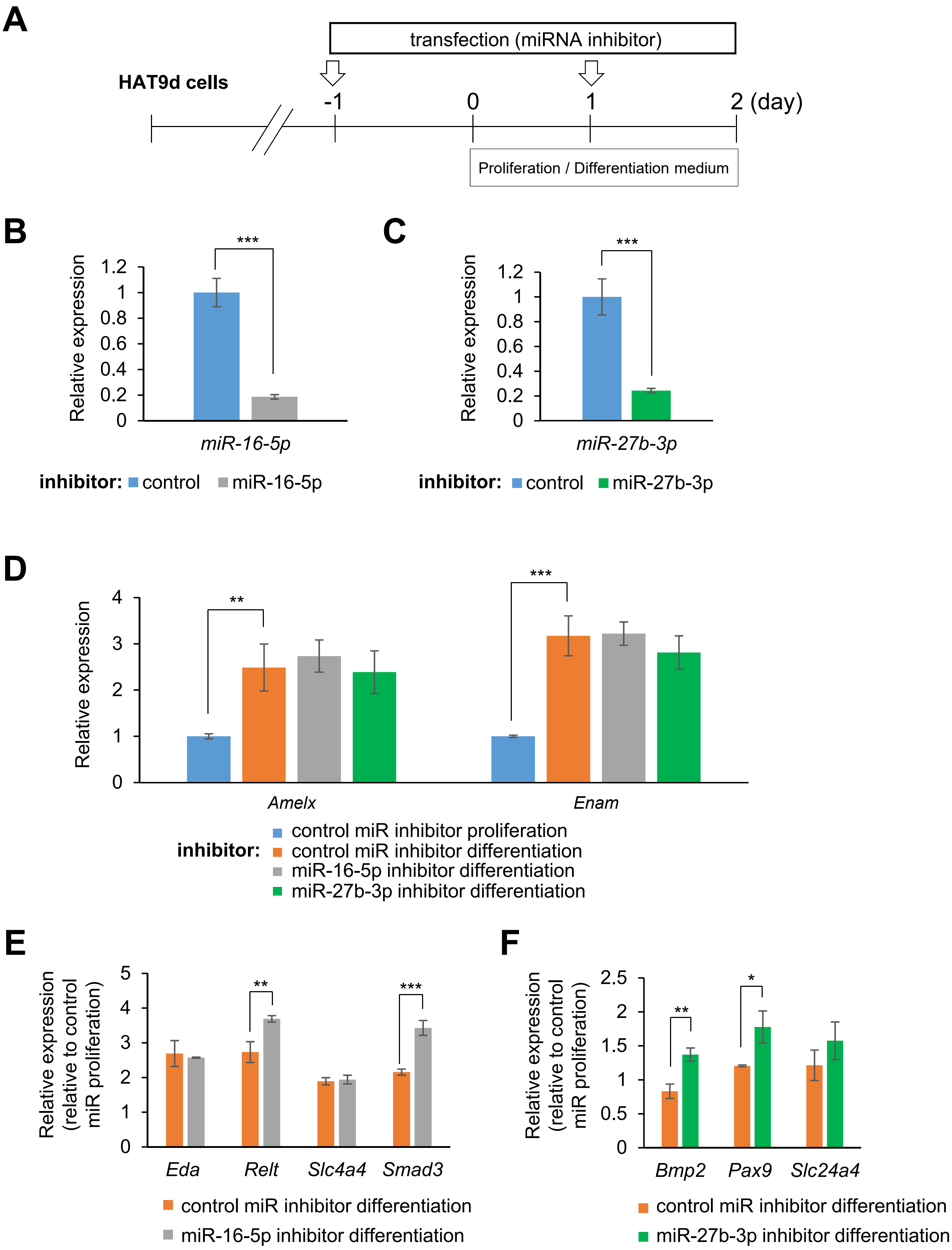

Supplementary Figure 8

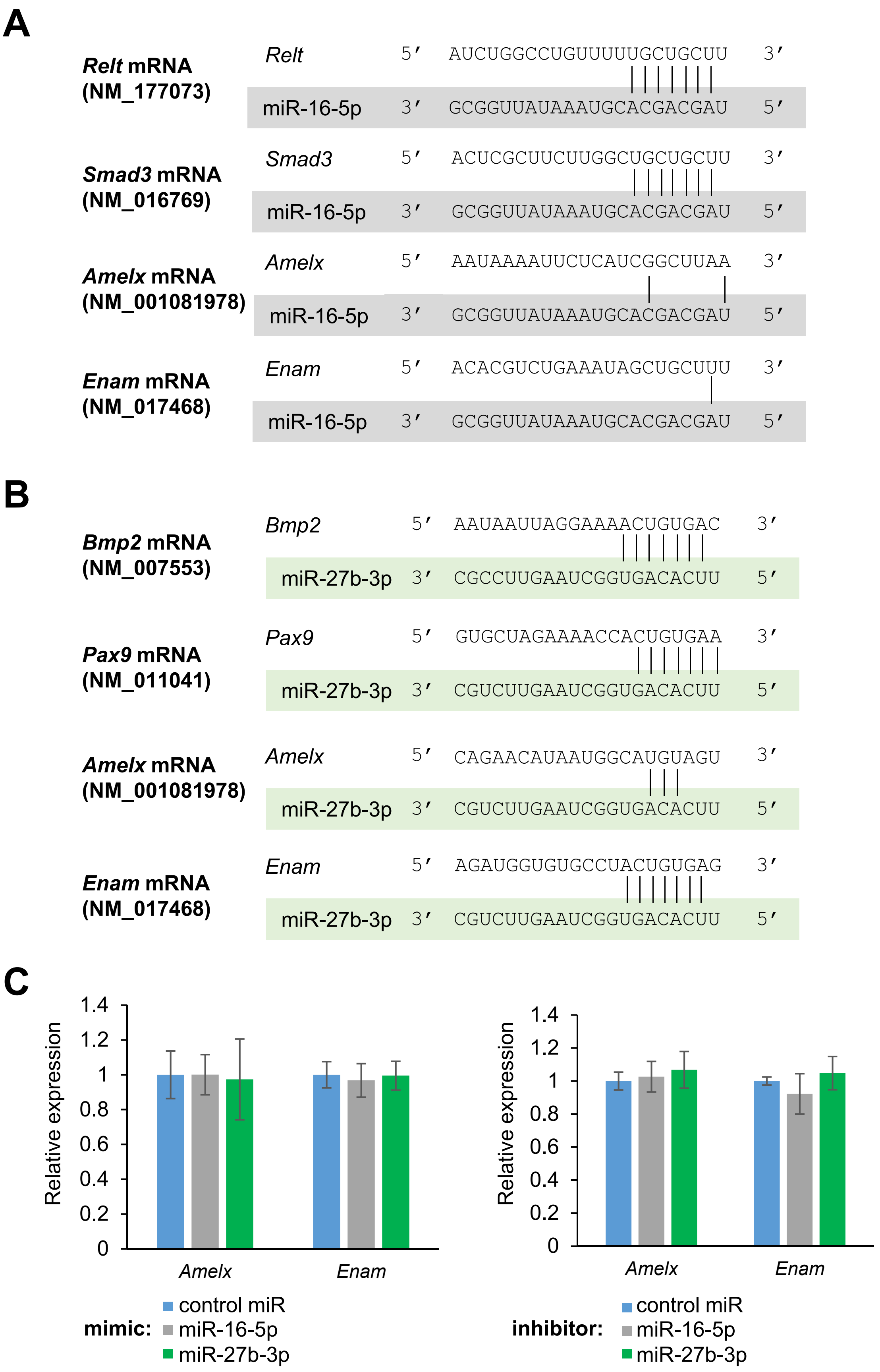

Supplement: Supplementary file 4 [file DataSheet1.PDF]
